# From structure prediction to function: defining the domain on the African swine fever virus CD2v protein required for binding to erythrocytes

**DOI:** 10.1128/mbio.01655-24

**Published:** 2024-12-17

**Authors:** Ana Luisa Reis, Anusyah Rathakrishnan, Vlad Petrovan, Muneeb Islam, Lynnette Goatley, Katy Moffat, Mai Tuyet Vuong, Yuan Lui, Simon J. Davis, Shinji Ikemizu, Linda K. Dixon

**Affiliations:** 1 The Pirbright Institute, Woking, Pirbright, Surrey, United Kingdom; 2 Radcliffe Department of Medicine, Weatherall Institute of Molecular Medicine, John Radcliffe Hospital, University of Oxford, Oxford, United Kingdom; 3 Graduate School of Pharmaceutical Sciences, Kumamoto University 13205 , Kumamoto, Japan; University of Pennsylvania, Philadelphia, Pennsylvania, USA; NRIVVaM, Pokrov, Russia

**Keywords:** African swine fever virus, immune evasion, EP402R, virulence factors, persistence, erythrocyte adhesion

## Abstract

African swine fever virus (ASFV) is a high-consequence pathogen posing a substantial threat to global food security. This large DNA virus encodes more than 150 open reading frames, many of which are uncharacterized. The *EP402R* gene encodes CD2v, a glycoprotein expressed on the surface of infected cells and the only viral protein known to be present in the virus external envelope. This protein mediates binding of erythrocytes to both cells and virions. This interaction is known to prolong virus persistence in blood thus facilitating viral transmission. The sequence of the extracellular domain of CD2v shows similarity with that of mammalian CD2 proteins and is therefore likely to feature two immunoglobulin (Ig)-like domains. A combination of protein structure modeling and extensive mutagenesis was used to identify residues mediating binding of transiently expressed CD2v to erythrocytes. The N-terminal Ig-like domain AGFCC′C″ β sheet was identified as the putative CD2v erythrocyte-binding area. This region differed from the putative CD58 ligand binding site of host CD2, suggesting that CD2v may bind to a ligand(s) other than CD58. An attenuated genotype I ASFV was constructed by replacing the wild-type *EP402R* gene for a mutant form expressing CD2v bearing a single amino acid substitution, which abrogated the binding to erythrocytes. Pigs immunized with the recombinant virus developed early antibody and cellular responses, low levels of viremia, mild clinical signs post-immunization, and high levels of protection against challenge. These findings improve our understanding of virus–host interactions and provide a promising approach to modified live vaccine development.

**IMPORTANCE:**

A better understanding of the interactions between viruses and their hosts is a crucial step in the development of strategies for controlling viral diseases, such as vaccines and antivirals. African swine fever, a pig disease with fatality rates approaching 100%, causes very substantial economic losses in affected countries, and new control measures are clearly needed. In this study, we characterized the interaction between the ASFV CD2v protein and host erythrocytes. The interaction plays a key role in viral persistence in blood since it can allow the virus to “hide” from the host immune system. We identified the amino acids in the viral protein that mediate the interaction with erythrocytes and used this information to construct a mutant virus that is no longer able to bind these cells. This virus induces strong immune responses that provide high levels of protection against infection with the deadly parental virus.

## INTRODUCTION

African swine fever virus (ASFV) causes a haemorrhagic disease, named African swine fever (ASF), in domestic pigs and wild boar with a case fatality that can approach 100%. ASFV is endemic or causes sporadic outbreaks in domestic pigs in most sub-Saharan African countries. In 2007, the disease spread to Georgia in the Caucasus region and subsequently to Russia and Eastern Europe entering the EU in 2014. Spread to China, the world’s largest pork producer, occurred in 2018, followed by spread to 17 additional Asian countries. In 2021, ASF was reported in the Dominican Republic and Haiti, raising the risk to neighboring countries in the Caribbean and Americas (World Organization for Animal Health [WAHIS]).

ASFV is a large DNA virus, the only member of the *Asfarviridae* family, and replicates in the cytoplasm of porcine monocytes and macrophages at an intermediate to late stage of differentiation (1). The virus genome codes for about 170 proteins, including many that are not essential for replication in cells but have roles in evading host defences. Viral particles can enter cells by several mechanisms, including clathrin-mediated endocytosis, macropinocytosis, and efferocytosis (2–7). The nucleoprotein core enters the cytoplasm after fusion of the internal envelope with that of late endosomes. Early gene transcription begins in the cytoplasm immediately after entry using the virus-encoded RNA polymerase, which is packaged in the nucleoprotein core with other enzymes and factors required for early transcription. The viral particle nucleoprotein core is surrounded by a core shell, internal envelope, and icosahedral capsid. Mature virions traffic from perinuclear virus factories to the plasma membrane and gain an external envelope during budding (8–11). Both the intracellular mature and extracellular enveloped virions are infectious. Of the approximately 70 viral proteins detected in purified virions, only one, CD2v encoded by the *EP402R* gene, was identified in the viral external envelope in a proteomics analysis (10).

The CD2v protein likely shares a similar domain structure but a low sequence similarity to host CD2. The CD2v protein, as CD2, contains a N-terminal signal peptide and very likely two immunoglobulin (Ig) like domains. The cysteine residues cross-linking the base of the second, membrane-proximal Ig2 domain, are a hallmark of host CD2 proteins, and are conserved in the CD2v protein (12, 13). A cytosolic domain following the single transmembrane domain in CD2v has varying numbers of proline-rich motifs and differs from host CD2 proteins in sequence and binding partners. The actin-binding protein SH3P7 (mAbp1) (14) and AP-1 adaptor protein (15) interact with the cytosolic tail of the CD2v protein, suggesting a role in protein transport or signaling pathways. The cytosolic domain was shown to be necessary for interaction with the stimulator of interferon (IFN) genes (STING) protein and the inhibition of IFN induction initiated by cGMP-AMP synthase (cGAS) activation (16). Additionally, CD2v was reported to interact with the colony-stimulating factor two receptor subunit alpha (CSF2RA) that activates the JAK2-STAT3 signaling pathway (17). The molecular basis of this interaction remains to be determined.

The host CD2 protein is expressed on the surface of T cells, NK cells, thymocytes, and dendritic cells (18, 19). The CD58 (or CD48 in rodents) ligand for CD2 is expressed on a variety of cells, including B cells, T cells, monocytes, granulocytes, and thymic epithelial cells. The interaction between CD2 on T cells and CD58 on antigen-presenting cells stabilizes the formation of cellular contact (20–22).

CD2v is required for the binding of erythrocytes to infected cells (hemadsorption [HAD]) and extracellular virions (13, 23). However, the CD2v protein is not essential for infection, and non-HAD isolates have been found in the field and obtained by targeted deletion of the *EP402R* gene. A large proportion (>90%) of ASFV particles in blood from pigs infected with HAD isolates is localized to the erythrocyte fraction (24), which may help to conceal viral particles and infected cells from components of the immune system thus reducing viral clearance (25). Previously, the expression of the CD2v protein by ASFV was shown to be important to enhance viral uptake by the soft tick vector *Ornithodoros spp*. Extending the period of viral persistence in blood would facilitate infection of the soft tick vector, and help to maintain a reservoir of infection in the sylvatic cycle in E. Africa (26). In the mammalian host, a role for CD2v in modulating T cell activity has been indicated since deletion of the *EP402R* gene (also named 8DR) from the ASFV genome was shown to abrogate the ASFV-induced inhibition of T cell proliferation in response to mitogens *in vitro* (25).

Evidence suggests that CD2v also has a role in cross-protection, since eight different immunotypes were defined based on the ability of attenuated viruses to induce cross-protection against challenge with diverse ASFV genotypes. This correlated with the ability of sera from immunized pigs to inhibit HAD. Since CD2v is required to induce HAD, its sequence may be used to predict cross-protective immunotypes. The C-type lectin homolog coded by the *EP153R* gene may also have a role in cross protection (27–29). CD2v and EP153R also contain T-cell epitopes (30, 31), thus cellular responses against these proteins may be important for protection. Additional support for a role of CD2v in protection comes from one study which showed that immunization with recombinant CD2v conferred partial protection of pigs against homologous challenge (32).

Little is known about the functional domains of CD2v and its interactions with host cell targets. Recently, a comparison between the CD2v sequences from HAD and non-HAD ASFV isolates identified the signal peptide and two glycosylation sites as important for binding to erythrocytes (33). However, the structural determinants of CD2v function have not been systematically explored. In the current study, we predicted the structure of the N-terminal CD2v extracellular domain for the ASFV genotype I Benin 97/1 strain. The Ig-like structural model of this domain, which we hereafter call Ig1, identified potential residues exposed on the surface of the protein that may interact with host proteins. We constructed mutant full-length *EP402R* genes by extensive “drastic” substitutions of single amino acid residues of the Ig1 domain. Subsequent assays utilizing transient expression in transfected cells identified mutations that abrogated binding of erythrocytes. We showed that the wild-type and mutant CD2v were expressed at the same level on the cell surface, supporting a direct role for these residues in binding to receptor(s) on the erythrocytes. Clustering of these residues on the AGFCC′C″ β sheet identified this as the putative CD2v erythrocyte-binding region. Two key residues, E99 and N108, are close to the edge of the CD58 binding site of human (h) CD2, but the other key residues lie outside this region, suggesting that CD2v may bind to different cellular ligands. These data provide an important step towards understanding the interactions of CD2v with specific receptors and cells. Our previous results showed that, following immunization of pigs with a genotype I Benin 97/1 virus from which the *DP148R* gene was deleted, the virus persisted in blood for greater than 50 days decreasing in titer over that period (34). Deletion of the *EP153R* gene in addition to *DP148R* did not reduce the period of virus persistence in blood, whereas deletion of the *EP402R* gene reduced the period of persistence in blood to 9 days. When both *EP402R* and *EP153R* genes were deleted in addition to *DP148R*, no viremia was detected after immunization, suggesting these that proteins may act synergistically. However, this triple-gene deleted virus induces low immune responses and reduced protection even after an additional boost (35). These results are summarized in Table S1. In our current manuscript, we replaced the wild-type *EP402R* gene with the gene expressing the non-HAD mutant CD2v protein with a single amino acid substitution E99 to R. The *DP148R* and *EP153R* genes were also deleted.

In the pigs immunized with this virus, low transient viremia and clinical signs were observed, but strong early antibody and cellular responses were induced. High levels of protection were observed following challenge with virulent parental virus. We recently reported similar results using an attenuated genotype II virus expressing a non-HAD CD2v (36); thus the approach appears to be generally applicable to diverse ASFV genotypes. The current study presents novel data greatly extending the mutagenesis of predicted surface residues of the CD2v protein to identify a putative domain involved in binding to red blood cells.

## RESULTS

### Predicted structure of the genotype I Benin 97/1 CD2v protein

**Fig 1:**
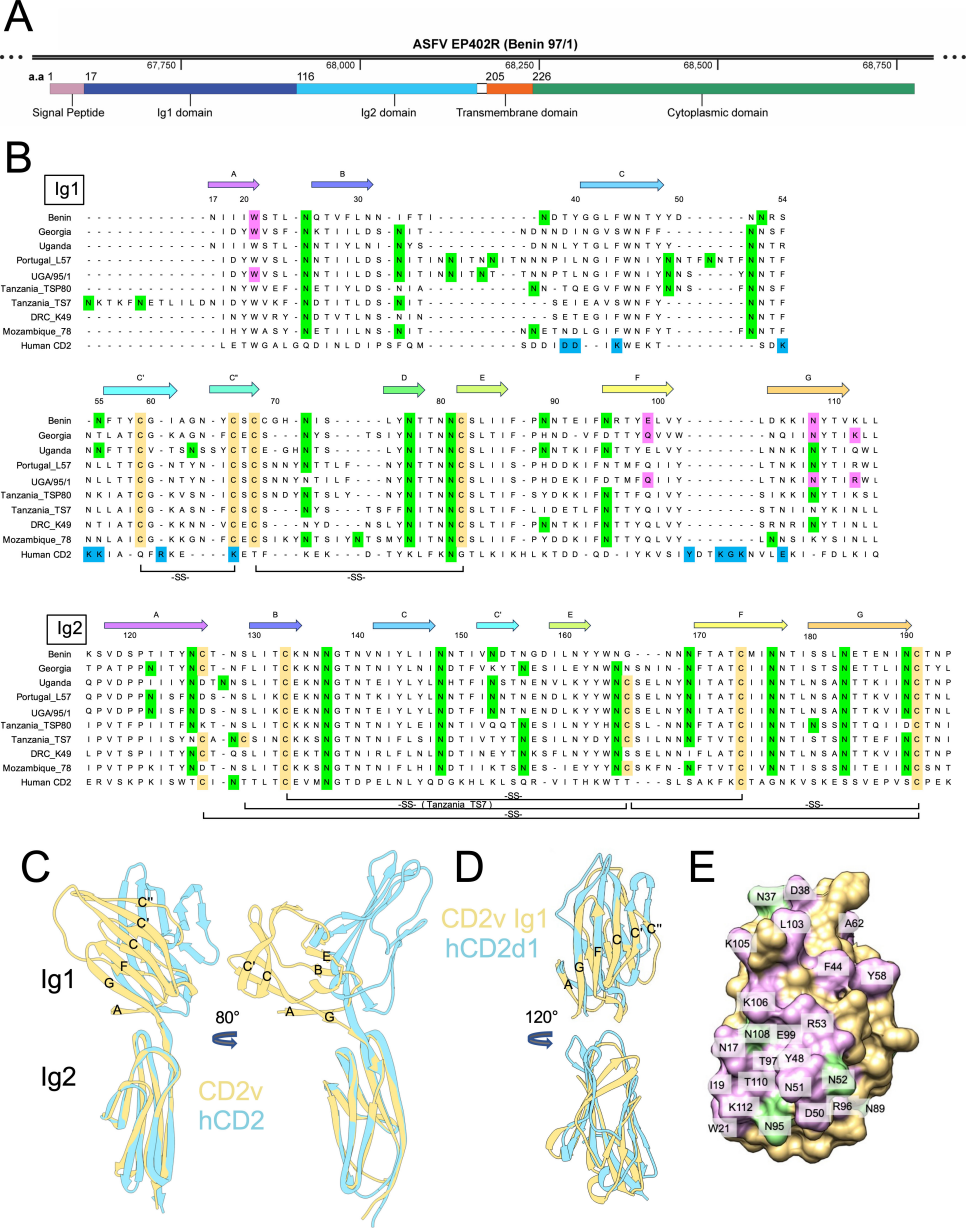
Modeling of the CD2v protein. (**A**) Domain structure of the genotype I Benin 97/1 encoded CD2v. The gene location is shown in the top panel. In the lower panel, the numbers represent the amino acids (aa) comprising each domain of the protein. (**B**) Alignment of the sequences of the extracellular domains of representative CD2v proteins and hCD2. Numbering refers to the Benin 97/1 CD2v sequence, and β-strands are labeled according to its structure. Putative glycosylation sites are colored green, and cysteine residues are colored orange with potential disulphide bonds shown underneath. Residues in magenta were identified in this study as involved in binding to erythrocytes and are highlighted here for future reference. Residues highlighted in blue in the human CD2 sequence are those known to mediate ligand binding. (**C**) Superimposition of the structure of the extracellular regions of hCD2 (blue), and the AlphaFold2-generated extracellular region of CD2v (Benin 97/1; orange), via their Ig2 domains. (**D**) Superimposition of the Ig1 domains of hCD2 (blue), and the AlphaFold2-generated extracellular region of CD2v (Benin 97/1; orange). The structures are viewed along an axis orthogonal to the AGFCC′C″ β sheet (top) and following a 120° anti-clockwise rotation along an axis in the plane of the page (bottom). (**E**) Surface representation of the Benin model (orange) showing the positions of the mutations (purple) and putative N-linked glycans (green).

The ASFV *EP402R* gene codes for the CD2v protein, which varies in length between ~370 and ~400 amino acids among different isolates mainly due to variation in the numbers of proline-rich repeats of the sequence PKPCPPP in the cytosolic region of the protein. Fig. 1A shows the domain organization of the encoded CD2v protein, including the N-terminal signal peptide, the two putative Ig domains, the transmembrane region, and cytosolic tail. The alignment of full-length extracellular regions of hCD2 and CD2v from representative ASFV immunotypes (Fig. 1B) highlights the predicted positions of the β-strands that form Ig1 and Ig2, but also, especially, the poor amino acid conservation between hCD2 domain 1 (d1), and the CD2v Ig1 sequences. Like mammalian CD2, the sequences indicate that Ig1 lacks the canonical disulphide bridge between cysteines of strands B and F in the IgV-like domains. Instead, CD2v is likely to have two unique disulfide bridges between β-strands C′ and C″, and between β-strands C″ and E in the IgV-like domain.

A plausible model of Ig1 of CD2v (genotype I Benin 97/1) was initially built using the program Modeller (37) and the structure of hCD2d1, and used as the basis of our mutational analysis. Subsequently, confidence in the model was strengthened with the use of AlphaFold2 (38). The main difference between the Ig1 domain of Benin CD2v and hCD2d1, lies in the positions of the C, C′, and C″ β strands, which, in the Benin structure, are rotated “backwards” from the plane of the AGFCC′C″ sheet by up to 73° (Fig. 1D). Models of the Ig1 domain of Georgia 2007/1 Genotype II and of the IgV-like domain of pig CD2 were also generated using AlphaFold2, and of the Ig1 domain of UGA/95/1 Genotype IX using Rosetta based on the Benin structure and compared with Benin Ig1 and hCD2d1 (Fig. S1AB). This analysis showed that Benin CD2v is, as expected, structurally more similar to the other viral variants than mammalian CD2 (i.e*.*, 41%, 46%, 12%, and 16% amino acid sequence identities for the Georgia, UGA/95/1, pig, and human CD2v/CD2 versus Benin, respectively), although there are conserved residues in all strands (except the C″ strand) likely to preserve the overall structure of the domains.

Figure 1C shows a superimposition, via Ig2, of the full extracellular region of hCD2 (blue; PDB: 1HNF) and the Benin 97/1 CD2v model (orange) predicted by AlphaFold2. The organization of the two structures is similar, except insofar as Ig1 of CD2v is rotated backward and downward versus hCD2d1 (the angle of rotation is 68°), placing the ligand-binding AGFCC′C″ sheet closer to the “top” of CD2v.

### Hemadsorption phenotype of the wild-type and mutant CD2v proteins transiently expressed in mammalian cells

To map the putative ligand binding site of CD2v, we adopted the approach used previously to identify residues important for ligand binding by CD2, i.e., by making single amino acid substitutions in the predicted surface residues of the CD2v Ig1 domain (39). Twenty-five single residues in the Ig1 domain were selected for mutation in the genotype I Benin 97/1 strain. This involved replacing negatively charged residues with positively charged ones and vice versa (D38R, D50R, R53D, R96D, E99R, K105D, K106D, K112D, and K115D). Additionally, some uncharged amino acids were replaced with a charged residue (N17R, I19R, W21D, F44D, Y48R, N51R, Y58R, A62R, Y65D, Y76D, N95R, T97R, Y102D, L103R, N108R, and T110R). Except for Y76 and Y102, all the residues were predicted to be exposed at the surface of the AGFCC′C″ β sheet according to our structural model (Fig. 1E). Y76 and Y102 are mostly buried (13% and 25% exposure of the side chains only, respectively) and located in β strands away from the predicted binding site. These two mutations were predicted to affect the overall structure of the Ig1 domain and were used as controls for folding.

**Fig 2:**
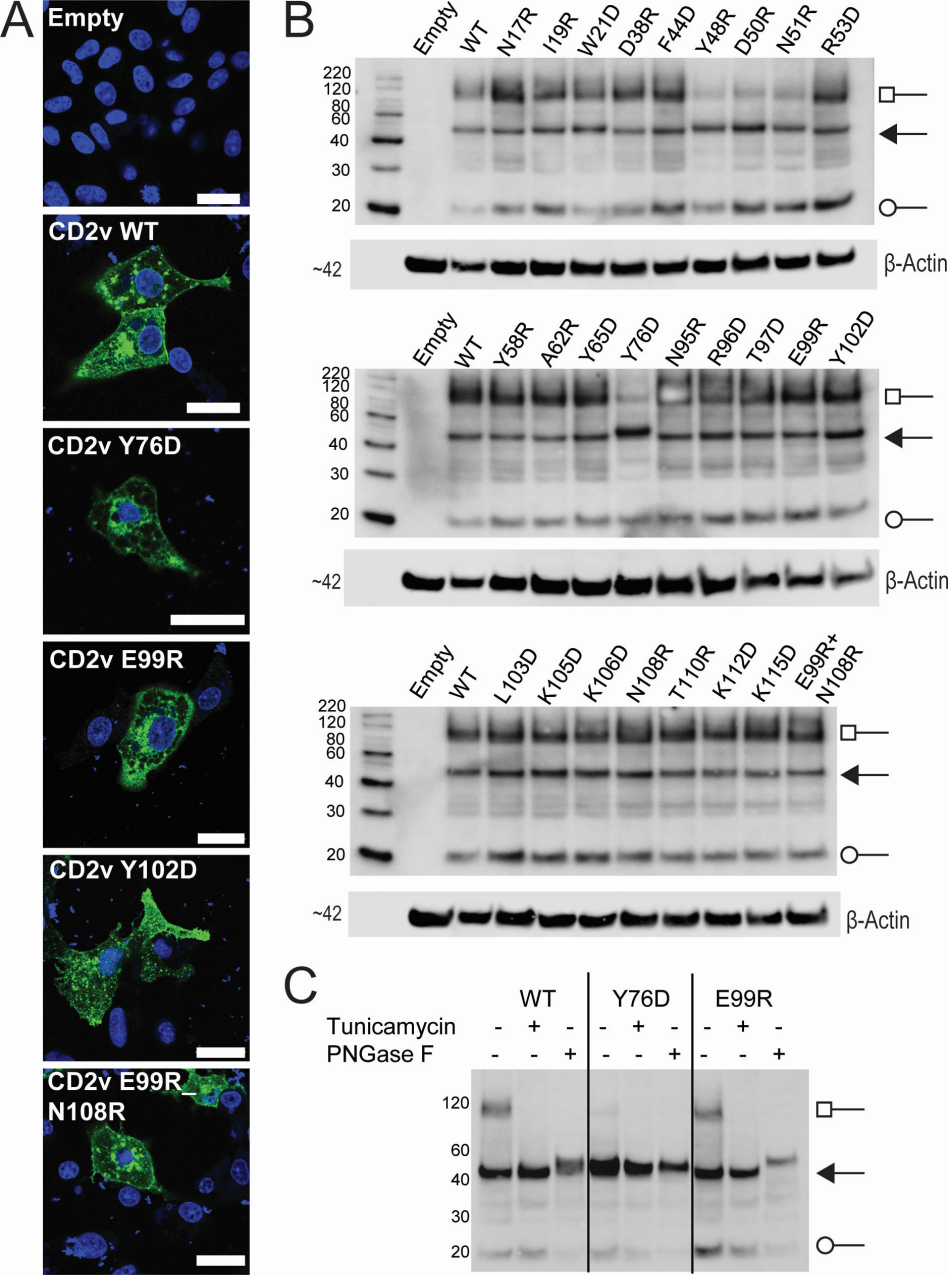
Expression of C-terminus HA-tagged CD2v in transfected cells. Vero cells were infected with MVA-T7 and transfected with plasmids expressing Benin 97/1 CD2v, wild-type (wt) or mutants with the indicated single or double amino acid substitutions. At 48 h post transfection, the cells were either fixed for immunostaining (A) or lysed (B and C) for Western blot analysis. (A) After fixation, cells were permeabilized and stained for HA tag expression (green) and counterstained with DAPI to show the nucleus (blue). Bars represent 25 μm. (B) Western blots of lysates from transfected cells probed with anti-HA and anti-β-actin antibodies. Molecular weight markers are shown on the left. Squares denote fully glycosylated CD2v, arrows denote full length CD2v, and circles denote cleaved CD2v. (C) Similar to panel B, but cells were treated with tunicamycin, or cell lysates were treated with PNGase F prior to Western blot.

Full-length *EP402R* complementary DNAs encoding the wild-type or mutant CD2v proteins with the selected single amino acid substitutions and a C-terminal HA tag were cloned into expression vectors under control of the T7 promoter. They were then transfected into Vero cells infected with the modified vaccinia Ankara (MVA) expressing the T7 RNA polymerase to drive high-level protein expression. This was assessed by immunofluorescence of fixed and permeabilized cells (Fig. 2A; Fig. S2) and by separation of cell lysates by SDS-PAGE followed by Western blotting (Fig. 2B). In fixed and permeabilized cells, the wild-type and mutant CD2v proteins produced a punctate staining pattern in the cytoplasm and at the cell periphery. Western blotting confirmed that the wild-type and mutant CD2v proteins, with the exception of Y76D, were expressed at similar amounts at the expected size for the full-length glycosylated proteins (~102 kDa) as well as an ~20 kDa C-terminal fragment described previously (40). In addition, bands were detected at the size similar to that predicted from the amino acid sequence (42 kDa). This was possibly due to delayed progression through the secretory pathway. The level of expression of this form of the protein was highest in the cells expressing the mutant Y76D, at the expense of the full-length glycosylated protein (~102 kDa) (Fig. 2B and C), suggesting that this species may comprise mis-folded proteins. Treatment of cells with tunicamycin to inhibit glycosylation or of cell lysates with N-glycosidase F (PNGase F; Fig. 2C) reduced the size of the band detected, confirming that the ~102 kDa band corresponds to a glycosylated form of CD2v. The 20 kDa band migrated at the same size in treated or untreated cells or extracts as expected for the C-terminal cleavage product from the cytosolic region (40).

**Fig 3:**
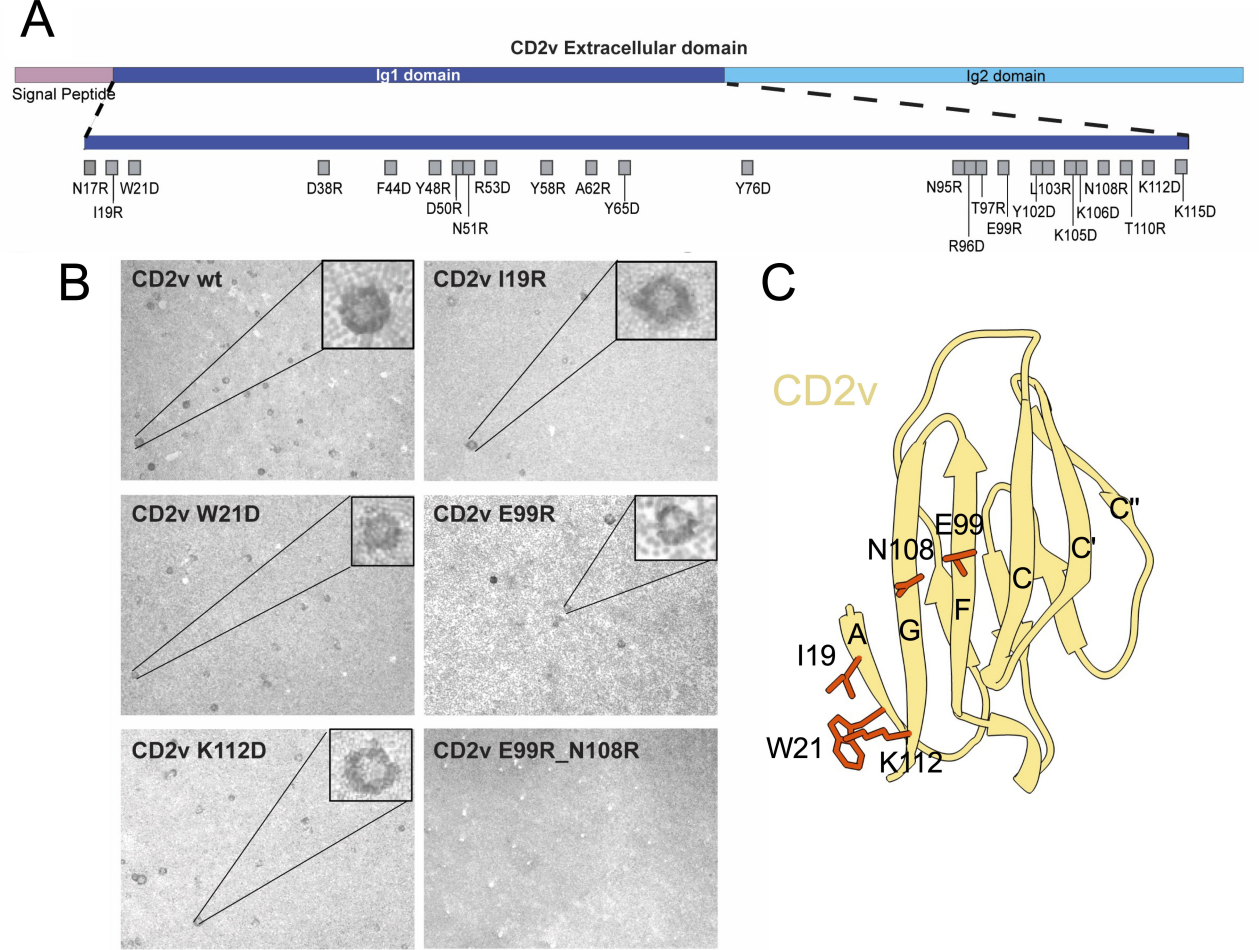
Mutagenesis and HAD in cells transiently expressing the wild-type or mutant CD2v. (**A**) Location of the amino acid substitutions within the CD2v Ig1 domain tested for HAD. (**B**) Vero cells were infected with MVA-T7 and transfected with plasmids expressing either the wild-type (wt) or mutant Benin 97/1 CD2v with the indicated single or double amino acid substitutions. After 48 h, erythrocytes were added, and the presence or absence of rosettes (HAD) was evaluated qualitatively. Representative fields are shown for the substitutions that resulted in reduced or no HAD, see Table 1 for more details (magnification of 5×). (**C**) The locations of the HAD-reducing mutations identified for the Benin 97/1, Georgia/2007/1, and UGA/95/1 strains, or the equivalent residues of Benin CD2v, are shown on the Benin CD2v model with the AGFCC′C″ β-sheets labeled based on the hCD2d1 structure.

Parallel transfections were carried out to evaluate the ability of the expressed proteins to bind to erythrocytes. Porcine erythrocytes were added to the MVA infected and transfected cells and wells were observed by light microscopy for formation of erythrocytes rosettes around the transfected cells. Experiments were repeated at least three independent times and the numbers of rosettes detected compared qualitatively. The results showed that most of the mutants except for those with mutations at positions Y76 (to D), E99 (to R), and Y102 (to D), induced similar number of rosettes to those induced by the wild-type CD2v (Fig. 3B; Table 1; Fig. S3). Additionally, mutations I19R and W21D resulted in a small, but consistent, reduction in rosette numbers. Of the amino acids affecting HAD, residue E99 is predicted to be exposed at the surface of the “F” β-strand, and therefore a putative ligand-binding site (Fig. 3C). A double mutant containing an extra mutation at position N108 (E99R + N108R) was then constructed and tested in a similar way. This was to ascertain the role of N108 in the binding mediated by E99. These two residues are predicted to be very close in the CD2v model, with N108 exposed at the surface of the “G” β-strand and potentially glycosylated. Although the single N108R mutation did not reduce HAD, the double mutation E99R + N108R, completely abolished binding of erythrocytes to transfected cells (Fig. 3B, lower right panel and Table 1).

**TABLE 1:** HAD phenotype of the different CD2v mutants tested

Benin 97/1 genotype I (immunotype 4)	HAD^*a*^	Georgia/2007/1 genotype II (immunotype 8)	HAD^*a*^	UGA/95/1 genotype IX (unknown immunotype)	HAD^*a*^
N17R	+++				
I19R	++				
W21D	++	W19D	+/−	W20D	+/−
D38R	+++				
F44D	+++				
Y48R	+++				
D50R	+++	N48R	+++		
N51R	+++	N49R	+++		
R53D	+++	F51D	+++		
Y58R	+++				
A62R	+++				
Y65D	+++				
Y76D	+/−				
N95R	+++	D92R	+++		
R96D	+++	T93D	+++		
T97R	+++	T94R	+++		
E99R	+	Q96R	++	Q112R	−
Y102D	+	W99D	++		
L103R	+++				
K105D	+++				
K106D	+++				
N108R	+++	N104R	+++		
T110R	+++	T106R	+++		
K112D	+++	K108D	−	R125D	−
K115D	+++	T111D	+++		
E99R + N108R	−	Q96R + N104R	−	Q112R + N121R	−

^*a*^ HAD relative to wild-type CD2v: +++, similar levels; ++, reduced levels; +, very reduced levels; +/−, occasional detection of rosettes, not observed in all repeat experiments; −, absence of HAD.

The significance of this putative erythrocyte-binding site was further tested using CD2v sequences encoded by two other ASFV strains (Table 1). Residues predicted to be exposed on the surface of genotype II Georgia 2007/1 (36) and genotype IX UGA/95/1 CD2v Ig1 and involved in binding to erythrocytes according to findings obtained with Benin 97/1 CD2v, were identified based on theAlphaFold2 models (Fig. S1AB). Thirteen single and one double amino acid substitution from genotype II, again covering the AGFCC′C″ β sheet, and three single and one double substitutions from genotype IX were chosen for mutation and tested for their impact on HAD. The Q96R (Georgia 2007/1) and Q112R (UGA/95/1) mutations, in the same positions as E99R of Benin 97/1, also reduced the number of rosettes, although less for the Georgia/2007 CD2v (36). The combined mutations Q96R + N104R or Q112R + N121R abolished HAD as also observed for the E99R + N108R mutation of Benin 97/1. For both Georgia 2007/1 and UGA/95/1 sequences, another single mutation, K108D or R125D respectively, also abolished HAD (Table 1). This contrasts with the mutation K112D in Benin 97/1 CD2v, which did not reduce HAD, despite being located at a similar position in the model (Fig. 3B and C).

We examined the conservation of these key residues by comparing 36 different CD2v protein sequences available in the public databases (Fig. S4). The residue W21 is fully conserved, and E99 is either E or conservatively substituted with Q (E: 42%, Q: 58%). N108 is very highly conserved (N: 92%, K: 8%) and likely to be glycosylated in 86% of the sequences. Finally, K112 is either N, Q, R, or K (K: 44%, N: 36%, Q: 8%, R: 11%) and so positively charged or polar. Analysis using the BLOSUM62 matrix (41, 42) revealed a sum of pairwise substitution scores of 4,590 (percentile: 0.67) for E99 and sum of pairwise substitution scores of 2,795 (percentile: 0.46) for K112, also indicating that these are conservative substitutions. Overall, a relatively high level of conservation was observed for the residues implicated in HAD by CD2v.

### Expression of the wild-type and mutant CD2v proteins on the cell surface

**Fig 4:**
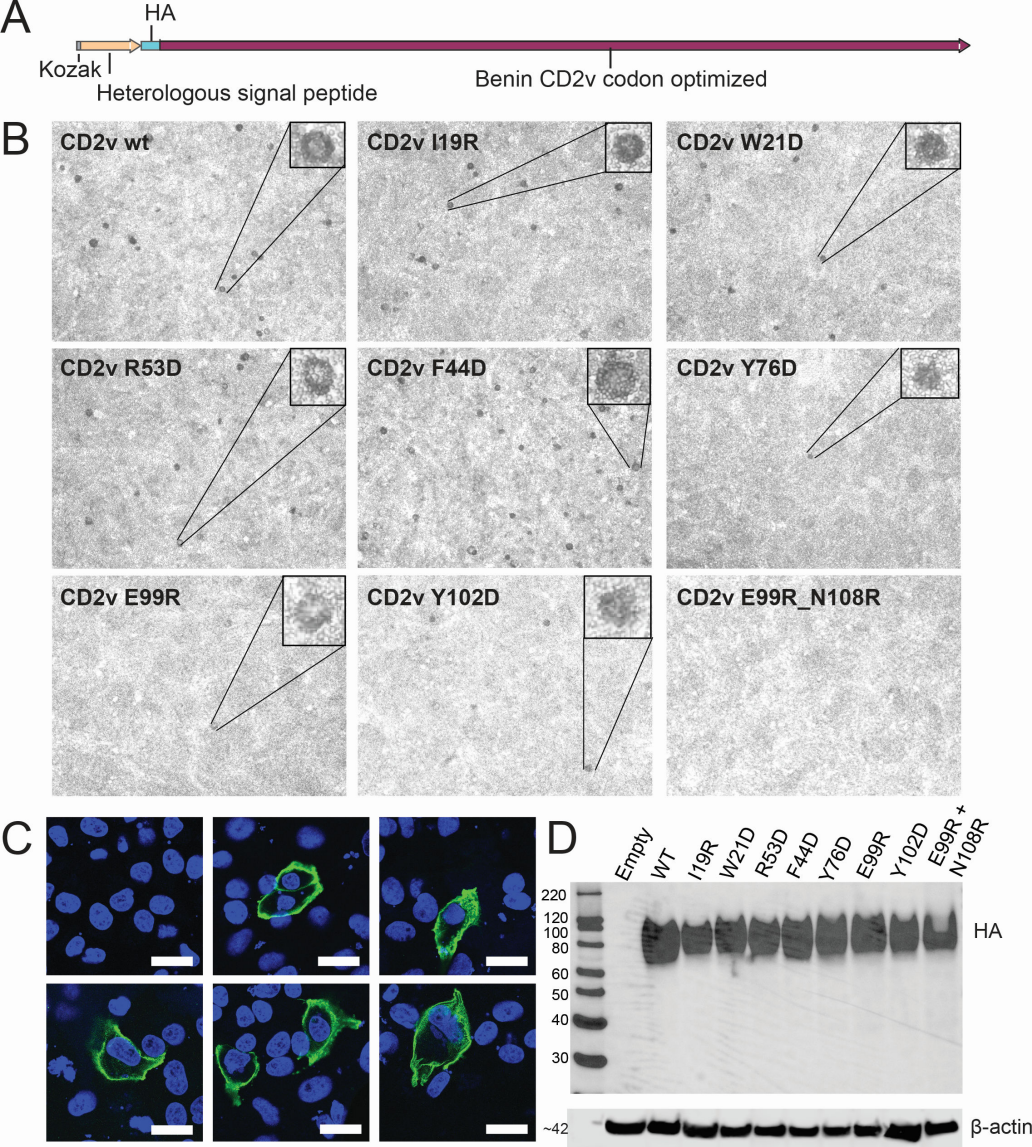
HAD evaluation and expression of N-terminus HA-tagged CD2v in transfected cells. (**A**) Construct designed to test cell surface expression of CD2v proteins. The HA tag is situated between a heterologous signal peptide and the CD2v extracellular domain (starting at position N16). (**B**) Vero cells were transfected with plasmids expressing wild-type (wt) or mutant Benin 97/1 CD2v with the indicated single or double amino acid substitutions. After 48 h, erythrocytes were added, and the presence or absence of rosettes (HAD) was evaluated qualitatively. Representative fields are shown (magnification of 5×). (**C**) Vero cells were transfected with the indicated CD2v wt or mutants as above and fixed for immunostaining of the cell surface using an anti-HA antibody (green). Nuclei are shown in blue. Bars represent 25 µm. (**D**) Western blots of lysates from transfected cells probed with anti-HA and anti-β-actin antibodies. Molecular weight markers are shown on the left.

To further investigate if the mutations affected binding of the CD2v protein to its ligand on erythrocytes, or instead may affect surface expression of the proteins, we designed wild-type and selected mutant *EP402R* genes with an HA epitope tag fused downstream of a heterologous signal peptide close to the N-terminus of the gene (Fig. 4A). Thus, we could confirm the expression of the proteins at the surface of non-permeabilized cells as well as in permeabilized cells.

Plasmids expressing these proteins under control of the CMV promoter were transfected into Vero cells, and formation of rosettes in the presence of erythrocytes was observed. The genes were codon optimized for mammalian cell expression, avoiding the need for MVA infection to boost expression. The results were similar to those described for the C-terminal tagged genes, as the mutants Y76D, E99R, and Y102D caused a marked decrease in rosette numbers and the E99R + N108R double substitutions abolished rosette formation (Fig. 4B). For the I19R and W21 mutants, the experiments were less consistent, and so they were repeated at least four times by two independent users. Table 1 reflects the consensus, with a slight decrease in rosette numbers for these mutants. Both the wild-type and CD2v mutant proteins were detected at the cell surface in non-permeabilized cells (Fig. 4C and Fig. S5). The N-terminal-tagged CD2v was detected as a broad band of 70 to ~102 kDa only (Fig. 4D), confirming that the 20 kDa band observed in cells expressing the C-terminal-tagged proteins (Fig. 2B) corresponds to the cleaved CD2v C-terminal fragment. Surprisingly, a 70 to 102 kDa band of the same size and intensity as detected in lysates from cells transfected with the wild-type or mutant CD2v proteins was also observed in lysates from cells transfected with the codon-optimized Y76D mutant. This might indicate that codon-optimization improved CD2v folding (43) and processing. Indeed, no band corresponding to the non-glycosylated full-length protein (42 kDa) was detected in any of the lysates. These results indicate that the observed impact of the amino acid substitutions on HAD was unlikely to be related to delayed protein progression through the secretory pathway to the cell surface. Instead, we predict these residues are involved in binding the ligand on the erythrocytes.

### Generation of the recombinant ASFV Benin∆DP148R∆EP153R-CD2v_mutantE99R

**Fig 5:**
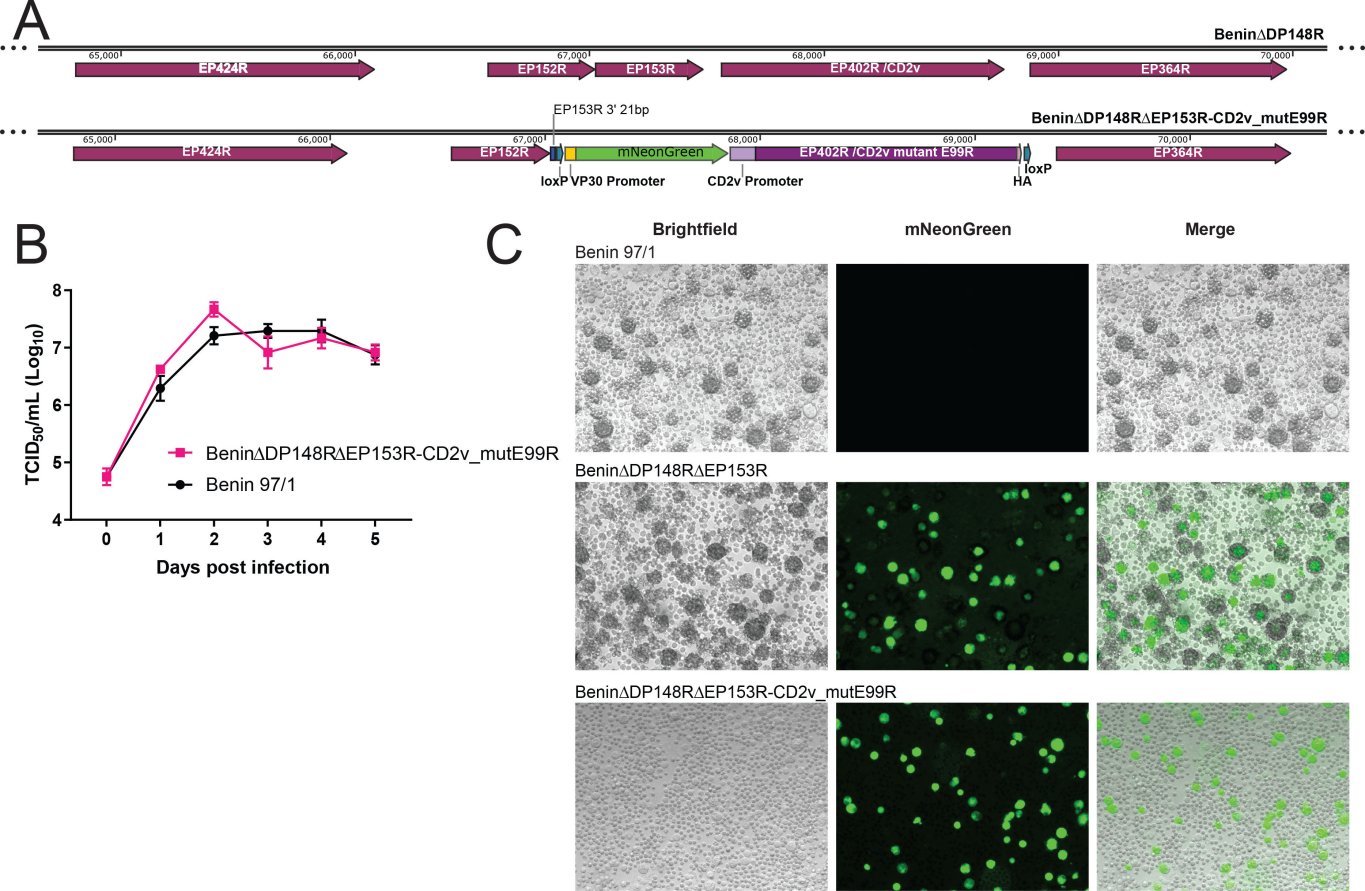
Characteristics of the recombinant ASFV BeninΔDP148RΔEP153R-CD2v_mutantE99R. (**A**) Schematics of deleting *EP153R* and replacing the wild-type *EP402R*, which encodes CD2v, with the single amino acid mutant CD2v at position E99. (**B**) Multistep growth curve of BeninΔDP148RΔEP153R-CD2v_mutantE99R and Benin 97/1 is shown over 5 days, and day 0 represents the inoculum. Two-way mixed effect ANOVA analysis with the Sidak’s multiple comparison test was performed to evaluate the difference between the groups over time. (**C**) *In vitro* infection of BeninΔDP148RΔEP153R-CD2v_mutantE99R is compared with wild-type Benin 97/1 and a recombinant ASFV, published previously (35) containing wild-type CD2v, but with *DP148R* and *EP153R* deleted (BeninΔDP148RΔEP153R).

Since residue E99 was predicted to be part of the CD2v ligand binding site, the E99R mutation was further characterized in the context of ASFV infection. A recombinant ASFV harboring the deletion of *EP153R* (genome position: 67051–67491) and expressing a CD2v protein with the E99R mutation (Fig. 5A) was purified as described in Materials and Methods. Subsequent sequencing of the *EP402R* gene confirmed the expected E99R mutation. Additional silent mutations at nucleotides 70 (T-C), 75 (T-C), and 450 (A-C), and one mutation at 419 (A-T) which altered amino acid asparagine (N, position 140) to isoleucine (I) were also detected. The latter was inadvertently created while introducing a restriction site. To confirm that the N140I mutation did not interfere with HAD, a codon-optimized construct with this single mutation was tested in transfected Vero cells as described above. As expected, the number of rosettes formed was similar to those observed in cells transfected with the wild-type CD2v (data not shown).

### Characterization of the ASFV Benin∆DP148R∆EP153R-CD2v_mutantE99R *in vitro*

PBMs were infected with the ASFV Benin∆DP148R∆EP153R-CD2v_mutantE99R or the wild-type Benin 97/1 isolates at a MOI of 0.01 to determine if the additional deletion of *EP153R* and mutagenesis of CD2v affected the ability of the virus to replicate *in vitro*. Total virus harvested from cells and supernatant at different days post-infection were titrated. The results showed no significant difference between the kinetics and the levels of viral replication of the recombinant virus and the parental Benin 97/1 isolate (Fig. 5B). Both viruses reached maximum titers of between 5 × 10^7^ and 10^8^ TCID_50_/mL, peaking at day 2 or 3 post-infection. In contrast to the wild-type virus and the BeninΔDP148RΔEP153R deletion mutant virus (35), no rosettes were observed following infection of PBMs with Benin∆DP148R∆EP153R-CD2v_mutantE99R (Fig. 5C).

### Immunization of pigs with the ASFV Benin∆DP148R∆EP153R-CD2v_mutantE99R and challenge with Benin 97/1

A group of six pigs (group B) were immunized and boosted on day 21 intramuscularly with 1 mL of Benin∆DP148R∆EP153R-CD2v_mutantE99R (group B) at 10^4^ TCID_50_. Three pigs, B1, B2, and B4, developed temperatures above 40.5°C at 5 days post-immunization (dpi), while the remaining three pigs had temperatures between 40.1°C–40.4°C. By 6 dpi, two pigs had temperatures below 40°C, and the other four below 40.3°C. Pigs B1, B2, and B4 also showed reduced eating for 1 day at 5 dpi. None of the six pigs showed any further clinical signs post-immunization or boost.

**Fig 6:**
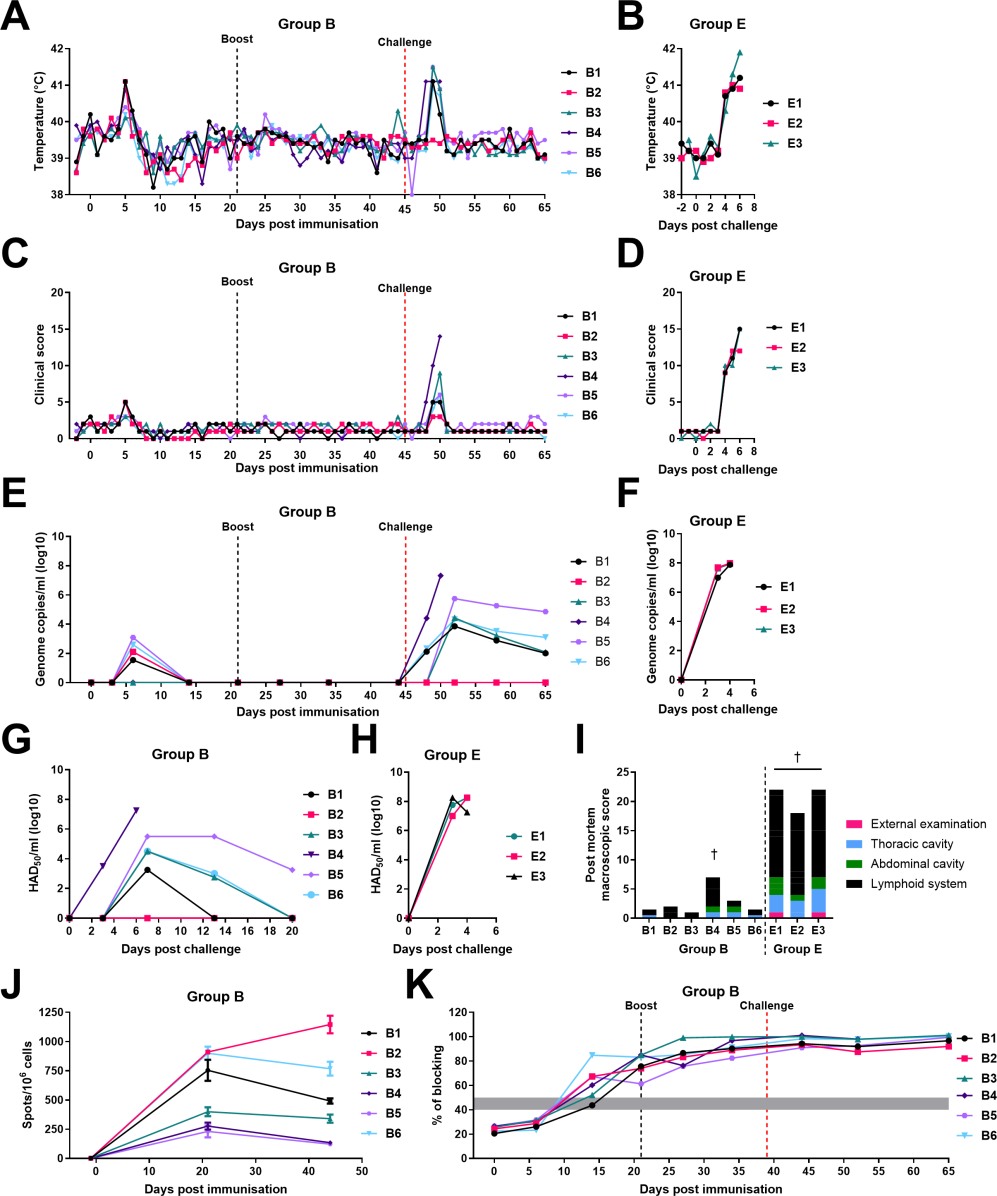
Temperatures, clinical scores, viremia, and immune responses of pigs immunized with the recombinant ASFV containing a mutant CD2v. Six pigs were immunized and boosted with BeninΔDP148RΔEP153R-CD2v_mutantE99R and subsequently challenged with Benin 97/1 (group B), and three pigs (group E) were used as non-immunized controls as described in reference 35. Daily (**A and B**) temperatures and (**C and D**) cumulative clinical scores were recorded. (**E and F**) Genome copies per mL of whole blood after immunization and challenge, measured via qPCR. The limit of detection is 1.59 × 10^2^ ASFV genome copies/mL. (**G and H**) Virus titers present in whole blood collected after challenge are shown for individual pigs as indicated. (**I**) Lesions observed during post-mortems were scored and displayed as a cumulative score. (**J**) The number of IFN-γ secreting cells in PBMCs of pigs before immunization, pre-boost, and pre-challenge was measured via ELISpot assays, and results are presented as mean number of IFN-γ cell spots per million PBMC. (**K**) ASFV-specific antibody responses against p72 were measured using a blocking ELISA. Results are presented as blocking percentage, where > 50% is considered positive.

The pigs in group B and a non-immunized group of three control pigs (group E) were challenged intramuscularly at 45 days post-immunization (dpi) with 10^3^ HAD_50_ of the ASFV virulent Benin 97/1 isolate. One pig in group B, B4, developed temperatures above 41.0°C for 3 days, from 3 days post-challenge (dpc), and was culled at the defined moderate humane endpoint on 5 dpc as it had stopped eating, and only moved when touched (Fig. 6A and C). Three pigs, B3, B5, and B6, had temperatures above 40.5°C for 2 days starting on 4 dpc; they also had reduced eating and were lethargic for 1 day, but then recovered. Pig B1 had a temperature above 40.5°C for 1 day (4 dpc). Pig B2 did not have increased temperatures but nevertheless had reduced eating and was lethargic for 2 days (4–5 dpc). Five pigs survived until the end of experiment (20 dpc). The post-mortem examination of pig B4 on 5 dpc revealed enlarged lymph nodes and mild hydropericardium and ascites. At the termination of the experiment on 20 dpc, only mild lesions were observed in surviving pigs, including mild enlargement of the submandibular lymph node, or the spleen and a very mild case of hydropericardium (Fig. 6I). The non-vaccinated, control pigs in group E challenged in parallel with group B have been previously described (35). They developed acute ASFV clinical signs between 2 and 4 dpc and were euthanized between 4 and 6 dpc at the moderate severity humane endpoint (Fig. 6B, D, and I).

### Levels of virus in blood

The levels of viral genome in EDTA blood samples collected throughout the experiment were quantified by qPCR. Low levels of viral genome were first detected in four pigs from group B (B1, B2, B5, and B6) at 6 dpi (10^1.5^–10^3.1^ genome copies/mL). After this, no viral genome was detected until after challenge. Viral genome was detected at 3 dpc in pigs B1, B4, and B6 (10^2.1^, 10^4.4^, and 10^2.3^ genome copies/mL, respectively). At the humane endpoint at 5 dpc, pig B4 had 10^7.3^ genome copies/mL. From 7 dpc to the end of the experiment, pigs B1, B3, B5, and B6 had low levels of viral genome in the blood; B1 from 10^3.9^ to 10^2.0^, B3 from 10^4.4^ to 10^2.1^, B5 from 10^5.7^ to 10^4.9^, and B6 from 10^4.3^ to 10^3.1^. No viral genome was detected in pig B2 after challenge. As expected, very high levels of virus genome (10^7^ to 10^8^ genome copies per mL) were detected in non-immunized group E pigs at termination (Fig. 6F) (35). Virus titers in blood, determined by hemadsorption, followed the same trend as for genome copies/mL (Fig. 6G). Pig B4 had detectable infectious virus at 3 dpc (10^3.5^ HAD_50_/mL), while the other pigs remained negative at this time point. At the humane endpoint on 5 dpc, pig B4 had 10^7.25^ HAD_50_/mL of blood. At 7 dpc the levels of infectious virus peaked on pigs B1, B3, B5, and B6 at 10^3.25^, 10^4.5^, 10 ^5.5^, and 10 ^4.5^ HAD_50_/mL, respectively. At the end of the experiment, at 20 dpc, only pig B5 had detectable infectious virus (10^3.25^ HAD_50_/mL). No viremia was detected in pig B2 after challenge (Fig. 6G). As expected, high levels of infectious virus were detected in non-immune challenged group E pigs (Fig. 6H).

### Immune responses to ASFV

The number of IFN-γ-producing cells stimulated by ASFV Benin 97/1 was evaluated using the PBMCs collected before immunization, pre-boost, and pre-challenge of the pigs (Fig. 6J). Before immunization, an ASFV-specific response was not detected in any of the group B pigs. Before boost, variable numbers of IFN-γ-producing cells were induced in the group B pigs (~231.3–911.3 SFC/10^6^ cells). At 44 dpi, the numbers of IFN-γ-producing cells dropped in five pigs to levels ranging from 122.5 to 767.5 spots/10^6^ cells. A similar dip in the number IFN-γ-producing cells has previously been observed in similar immunization studies with Benin gene-deleted attenuated viruses (35). Pig B2 had an average of 1,146.3 SFC/10^6^ cells pre-challenge, an increase from 911.3 SFC/10^6^ cells observed pre-boost.

The antibody response to p72 protein in serum samples collected at the indicated days (Fig. 6K) post-immunization and challenge was measured using a commercial blocking ELISA. Four of the pigs immunized with Benin∆DP148R∆EP153R-CD2v_mutantE99R showed antibodies above the detection cutoff by 14 dpi, while two remained doubtful. However, by day 21, all six pigs had levels of anti-p72 antibodies above the threshold and reached a plateau before challenge.

## DISCUSSION

A lack of knowledge of ASFV host interactions and evasion of host defences limits understanding of the molecular determinants of pathogenesis and hinders the development of modified live vaccine candidates. Here, we describe experiments that have advanced understanding on the role of the ASFV CD2v protein in host interactions, specifically by mapping the domain and critical residues required for binding of the expressed protein to erythrocytes. CD2v is expressed on the plasma membrane of the infected cells at late time points post-infection (23) and incorporated into the outer envelope of the viral particle during virus budding from the cell (9). The CD2v protein is not required for virus replication since the *EP402R* gene, coding for CD2v, can be interrupted by frame shift mutations on the genome of some naturally attenuated viral isolates (for example OURT88/3, NHV68, Lv17/WB/Rie1) (44–46) and has been deleted from the genomes of several virulent strains. The deletion or interruption of the gene did not reduce viral replication in macrophages (35, 47). Deletion of the *EP402R* gene from the genome of virulent isolates of genotype VIII Malawi and genotype II Georgia (25, 47) did not attenuate the virus in pigs, although deletion from the virulent genotype I virus BA71 resulted in virus attenuation (48). The varying impact of deleting or interrupting the *EP402R* gene is likely to result from variation in the other genes encoded by different isolates.

The CD2v protein has important roles in viral replication in its hosts. Firstly, it is required for binding of erythrocytes to infected cells and extracellular virus particles. This can aid viral dissemination and persistence in blood in the swine hosts (24, 25). We showed previously that deleting the *EP402R* gene from a moderately attenuated ASFV, BeninΔDP148R, dramatically reduced the period of viral persistence in blood but did not significantly reduce the moderate clinical signs observed (35). Expression of the CD2v protein is required for efficient uptake from blood by the arthropod vector of ASFV, soft ticks of *Ornithororos* spp. (26). Thus, longer-term persistence of virus in blood would favor the maintenance of the virus in its sylvatic cycle in east and south Africa. The advantages conferred to the virus by expression of the CD2v protein are offset by disadvantages to the virus. It is the only virus protein detected on the external envelope of the virus particle and thus likely to be exposed to the host humoral response. The CD2v protein is heavily glycosylated potentially hiding important epitopes recognized by antibodies. The CD2v and EP153R proteins are among the most variable ASFV proteins and indicated to be involved in protection. Antibodies generated in pigs by immunization with attenuated isolates can inhibit HAD induced by infection of cells *in vitro* with the homologous strain. The ability to inhibit HAD induced by infection of cells with heterologous strains correlated with the ability of the attenuated strain to induce cross-protection against the heterologous strain (27, 29, 31, 49). Immunization of pigs with recombinant CD2v protein was shown to induce partial protection (32). T cell epitopes have been identified encoded by the CD2v cytoplasmic domain (30). An additional immunomodulatory role for CD2v was suggested by experiments showing that its expression was required for the *in vitro* inhibition of lymphocyte proliferation in response to mitogens (25).

In this study, we investigated which residues on the ASFV CD2v protein are required for binding of the protein, transiently expressed in mammalian cells, to erythrocytes. The aim was to establish if the binding domain was similar or differed from that of host CD2. This would provide a first indication of both the binding domain and the identity of a possible cellular receptor for CD2v. We first generated a model of the Benin 97/1 CD2v protein based on the structure of the same domain from hCD2. Confidence in the model was further increased through the use of AlphaFold2. Surface residues in the ligand-binding domain Ig1 were predicted and mutations to alter the charge of single amino acids were introduced into the full-length gene. Transient expression of the wild-type or mutated CD2v proteins in mammalian cells identified which mutations reduced the binding of erythrocytes to the expressed CD2v proteins. The results showed that the E99 residue is likely to be critical to the binding to erythrocytes. An E99R construct with an epitope tag fused C-terminally to the signal peptide and ahead of the ligand-binding domain showed that this mutation did not reduce cell surface expression of CD2v in transfected cells, indicating a probable role of the residue in the interaction with its putative receptor. The importance of this residue was further confirmed by mutagenesis of CD2v proteins encoded by other ASFV strains, with the mutations Q96R (in Georgia/2007, genotype II) and Q112R (in UGA/95/1, genotype IX) also reducing or abolishing HAD, respectively. Other residues exposed at the surface of the predicted AGFCC′C″ β sheets were also shown to contribute to binding to erythrocytes, namely I19, W21, N108, and K112, although the latter only in Georgia/2007 and UGA/95/1 strains. Interestingly, N108 seems to cooperate with E99, since the double mutations E99R and N108R completely abolished hemadsorption, while the single N108R mutation did not impact erythrocytes binding. Overall, these residues are relatively well conserved across the isolates, which would imply that they form part of a shared contiguous binding site across the A, G, and F β strands. Curiously, however, in >80% of cases, N108, located towards the center of this region, is likely to be glycosylated, which would be expected to prevent binding. The location of the I19, W21, and K112 at the base of Ig1, also raises the possibility that Ig2 contributes to binding. For now, the full extent and nature of the CD2v erythrocyte-binding site remains uncertain. What is clear is that there is little involvement of the CC′C″ strands, in contrast to the interaction of hCD2 and hCD58 (21), suggesting that CD2v might bind to a different ligand(s) (Fig. S1C). Evidence that CD2v interacts with the host CD58 is very scarce. In cells co-transfected with both CD2v and porcine CD58, the proteins were shown to co-immunoprecipitate (50). Nonetheless, since both proteins were expressed in the same cell, it cannot be concluded that this reflects a role for the interaction in binding to erythrocytes. Others have recently demonstrated that the mutation of the amino acid N104 in Arm/07 CD2v (corresponding to N108 in Benin/97) only abolishes HAD when an additional mutation at N79 is present (33), perhaps indicating a role for this residue, and hence glycosylation, in CD2v structural stability. This is not surprising since glycosylation is critical for the orientation and presentation of CD2 extracellular domain (51), and glycosylation may play a similar role for CD2v function. The authors also showed that the signal peptide was important, and that an altered N-terminal sequence of the CD2v protein from natural non-HAD isolates OURT88/3 and NHP68 abrogated HAD (33) possibly by affecting the folding or cell surface expression of the CD2v protein.

Identification of the residues on CD2v required for binding to erythrocytes enabled us to construct the recombinant ASFV, which expressed a mutant CD2v protein that did not induce the hemadsorption phenotype. Our hypothesis was that insertion of this mutant *EP402R* gene into the attenuated triple-gene deletion virus BeninΔDP148RΔEP153RΔCD2v to generate BeninΔDP148RΔEP153R-CD2v_mutE99R would increase immunogenicity of the virus but retain the attenuated phenotype and low viremia and persistence of virus in blood. We showed that the modification did not reduce the ability of the virus to replicate in primary macrophages over a multi-step growth curve compared with the wild-type Benin 97/1. We could not confirm the expression of both the wild-type and mutant CD2v proteins in ASFV-infected cells due to the absence of a suitable antibody. Nonetheless, infection of porcine bone marrow cells with BeninΔDP148RΔEP153R-CD2v_mutE99R showed a reduced hemadsorption phenotype since erythrocytes rosettes were rarely observed around the infected cells compared with cells infected with viruses expressing the wild-type CD2v protein. Immunization of pigs with the BeninΔDP148RΔEP153R-CD2v_mutE99R confirmed that the attenuated phenotype detected previously in pigs immunized with BeninΔDP148RΔEP153RΔCD2v was retained. However, a transient increase in temperature for 1 day at 5 dpi above 40.5°C was observed in three pigs immunized with BeninΔDP148RΔEP153R-CD2v_mutE99R. In our previously published experiment, which was carried out in parallel with this one, no clinical signs were observed in pigs post-immunization or boost with BeninΔDP148RΔEP153RΔCD2v. Low levels of viral genome were detected, 10^1.5^–10^3.1^ genome copies per/mL, in blood on 1 day post-immunization (6 dpi) from four pigs immunized with BeninΔDP148RΔEP153R-CDv_E99Rmut. In contrast, no viral genome was detected in the blood from the pigs immunized with BeninΔDP148RΔEP153RΔCD2v. Thus, the insertion of the *EP402R* gene expressing the non-HAD CD2v E99R mutant protein slightly increased the viral replication in pigs compared with the BeninΔDP148RΔEP153RΔCD2v virus.

The pigs immunized with BeninΔDP148RΔEP153R-CD2v_mutE99R showed early antibody responses measured by a blocking ELISA against the major capsid protein p72 and cellular responses measured by IFN-γ ELISpot assays. The increase in the immune response compared with the one following BeninΔDP148RΔEP153RΔCD2v immunization may be due to slightly increased replication of the virus as described above. Protection against challenge with parental virulent virus Benin 97/1 showed BeninΔDP148RΔEP153R-CD2v_mutE99R induced protection of five out of six pigs using a moderate severity human endpoint. BeninΔDP148RΔEP153RΔCD2v induced low immune responses but after a second boost six out of eight pigs were protected against virulent virus challenge (35) (Table S1).

Natural non-HAD attenuated isolates described include genotype I strains from Portugal OURT88/3 and NHP68 (52, 53), and genotype II strains from Latvia (54) and China (55) . Immunization of pigs with, for example OURT88/3 strain, induced protection against challenge with related virulent isolates of up to 100%, depending on the dose and route of immunization (56). Low or no levels of viral genome were detected in blood, and strong antibody and cellular responses were generated. However, OURT88/3 and other natural non-HAD strains can cause a chronic form of ASFV disease characterized by skin and joint swellings, necrotic skin lesions, and other signs (53, 57), making these unsuitable for vaccine development. Deletion of additional genes from OURT88/3 did not reduce the signs of chronic disease but reduced protection induced (58). The mechanisms involved in induction of chronic disease forms are not known. We did not observe this type of chronic disease in any of the pigs immunized with the gene deleted and modified attenuated strains described in this or our previous studies. Thus, these strains are worthy of further safety and efficacy evaluation.

In summary, our results provide detailed information on the residues required for binding of the ASFV CD2v protein to erythrocytes and indicate a different ligand other than CD58 is involved. Further research will be required to confirm the ligand for CD2v and to determine the spectrum and phenotype of host cells to which CD2v binds and the impact of the binding. Our results also reveal a novel route to develop safe and efficacious live attenuated vaccines that is applicable to other ASFV genotypes, as we have recently demonstrated for the genotype II Georgia 2007/1 strain (36). Whether this strategy can be applied to the development of live attenuated vaccines against other arthropod-borne viruses (arboviruses) remains to be elucidated. Host viremia plays a crucial role in arboviruses’ transmission, both in terms of magnitude and duration (59, 60). Similarly to ASFV, many arboviruses bind to erythrocytes, although likely using different host interacting partners. Therefore, it is tempting to speculate that this approach might be an additional tool to control these emerging viruses threatening both human and animal health. Our study also provides an example on how to use recent advances in protein structure prediction tools to guide functional studies and ultimately uncover new aspects of virus–host interactions.

## MATERIALS AND METHODS

### Cells and viruses

African green monkey kidney epithelial cells (Vero, ECACC 84113001) were cultured in DMEM (Thermo Fisher Scientific) supplemented with 10% fetal bovine serum (FBS) (Life Science Production) and 1% penicillin–streptomycin (pen-strep) (Thermo Fisher Scientific). These cells were utilized in the *in vitro* assays described below. Porcine bone marrow cells (PBMs) were obtained as previously described (61). Wild boar cells (WSL-R) were obtained from the Friedrich Loeffler Institute and were grown in ZB28 medium (50% Ham’s F12 Nutrient Mix medium, 50% IMDM, both from Thermo Fisher Scientific) supplemented with 10% FBS, 1% pen-strep, and 1% L-glutamine (Thermo Fisher Scientific). WSL-R cells were used for initial infection with ASFV and transfection with transfer plasmids to enable homologous recombination. Subsequently, the isolated PBMs were used to purify the recombinant ASFV harvested from the WSL-R cells (see below). PBMs were used to grow and titrate all viruses used in this study. The ASFV Benin 97/1 and Benin∆DP148R isolates have been described elsewhere (34, 44). Viral titrations were carried out in quadruplicate by hemadsorption assay (HAD_50_/mL) or by end-point titers based on expression of mNeonGreen (TCID_50_/mL). Titers were calculated using the Spearman and Kärber algorithm.

### Mutation of the *EP402R* gene

Mutations of the *EP402R* gene were designed to substitute single amino acid residues in the predicted ligand-binding Ig1 domain. The Ig1 of CD2v was initially modeled using the protein modeling program Modeller using the hCD2d1 structure. Subsequently, an Ig1–Ig2 structure of CD2v was built using AlphaFold2. The CD2v/CD58 complex was also modeled, using the human CD2/CD58 complex structure (PDB ID: 1qa9) and simple superposition. Initial mutational candidates were selected from the CD58 binding area on CD2v, and then expanded toward the bottom of Ig1.

The full-length *EP402R* gene from the Benin 97/1 isolate (AM712239) fused with a C-terminal HA was cloned into pcDNA3 under control of the T7 or CMV mammalian promoter. The selected mutations were introduced in the *EP402R* sequence using the Q5 Site directed mutagenesis kit (New England BioLabs) following the manufacturer’s instructions. The codon-optimized wild-type and mutant *EP402R* sequences were obtained from Genscript already cloned into pcDNA3.

### Transient expression of the CD2v

To drive expression of CD2v cloned under the control of a T7 promoter, defective replicating viral vector, MVA expressing T7 RNA polymerase (MVA-T7) (62) was used to infect Vero cells before transfection with the plasmids expressing wild-type or mutant versions of CD2v, fused with an HA epitope tag at the C-terminus. In other experiments, the wild-type or mutant versions of CD2v were codon optimized for mammalian expression and were constructed with the HA epitope tag at the N-terminus downstream of a signal peptide. In some experiments, tunicamycin was added at 1 µg/mL. Transfections were performed using the TransIT-LT1 DNA Transfection Reagent (Mirus Bio LLC, USA), as per the manufacturer’s guidelines. Transfected cells were generally incubated for 48 h before proceeding with the following assays: hemadsorption assay, immunofluorescence staining, and Western blotting, all which are described below.

### Hemadsorption assay

Forty-eight hours after transfections with plasmids expressing the wild-type or mutant versions of CD2v, cells were detached using 0.05% Trypsin-EDTA (Thermo Fisher Scientific, UK) and resuspended in complete DMEM. Porcine erythrocytes obtained from heparinized blood were washed twice with PBS before adding onto detached Vero cells to induce HAD. Cells were incubated for another 16 h, and formation of rosettes was observed and imaged using a Leica DMi1 microscope attached to a Leica MC170 HD camera.

### Labeling of transfected cells for immunofluorescence

Vero cells transfected with plasmids expressing wild-type or mutant versions of CD2v, were washed twice with PBS, fixed with 4% paraformaldehyde, and permeabilized with 0.2% Triton X-100 when indicated before blocking with 0.5% bovine serum albumin (BSA) in PBS. The cells were immunostained with rat anti-HA high affinity antibody (Sigma, UK), followed by staining with goat anti-rat IgG (H+L) highly cross-adsorbed Alexa Fluor 488 secondary antibody (Invitrogen). Cell nuclei were counterstained with DAPI, and coverslips were mounted on Vectashield antifade medium (Vector Laboratories, UK) on glass slides. The cells were imaged via the Leica SP8 CLSM, upright microscope.

### Construction of the recombinant BeninΔDP148RΔEP153R-CD2v_mutE99R ASFV

Transfer plasmid p∆EP153R-CD2vE99R-PP30mNeonGreen was synthesized commercially (Genscript, USA). The plasmid contains (i) 806 bp left flanking region of EP153R (positions 66245 to 67050); (ii) sequences of a fluorescent reporter protein, mNeonGreen, under the control of the ASFV p30 promoter (63); (iii) wild-type or mutant version of the *EP402R* gene expressing the CD2v protein with an amino acid mutation at position 99 (E → R); and (iv) 805 bp right flanking sequence of CD2v (positions 68776 to 69580) (Fig. 5A).

The recombinant ASFV BeninΔDP148RΔEP153R-CD2v_mutE99R was produced by homologous recombination in WSL-R cells between a transfer plasmid, p∆EP153R-CD2vE99R-PP30mNeonGreen, transfected using the TransIT-LT1 reagent and the BeninΔDP148R virus infected at MOI 2. The recombinant virus harvested from infected and transfected WSL-R cells was purified from the parental virus by three rounds of single mNeonGreen fluorescent cell isolation into PBMs using a BD Bioscience FACS Aria Fusion with DIVA 9 software. This was followed by a further purification by two rounds of limiting dilutions in PBM cells (61). Sanger sequencing of PCR fragments generated across the site of deletion confirmed the expected deletion, mutation(s) and site of the reporter gene insertion.

### Whole genome sequencing of BeninΔDP148RΔEP153R-CD2v_mutantE99R

Virus was grown in purified PBMs and harvested 4 days post-infection. The cells and supernatants were separated via centrifugation at 1,000 × *g* for 10 min, and only the supernatant containing extracellular virus was used for whole genome sequencing. The virus was first concentrated via ultra-centrifugation at 13,000 × *g* for 1.5 h at 4°C and then treated with DNase I. Viral DNA was extracted using the MagAttract HMW DNA kit (Qiagen), and DNA libraries were prepared using the Illumina DNA prep kit (Illumina). DNA sample was sequenced on MiSeq instrument using a 600 cycle v3 cartridge, and a total of 4,751,144 sequence reads were obtained. Using the GS-based variant analysis module in SeqMan NGen (DNASTAR Lasergene 17) and the ASFV Benin 97/1 (accession number: AM712239) as reference genome, 87.4% of the sequences were assembled with a median coverage of 6325.33.

The complete genome sequence of BeninΔDP148RΔEP153R-CD2v_mutantE99R confirmed the deletions of genes *DP148R* and *EP153R*, along with the replacement with reporter markers GUS and mNeonGreen, respectively. The mutation of amino acid E to R, at position 99 in the CD2v protein was also confirmed. An additional deletion of 3,353 bp from genome positions 24,459 to 27,811, resulted in the partial deletion of the 5′-ends of the *MGF360-12L* and *MGF505-2R* genes and complete deletion of the *MGF360-13L* and *−14L* genes. This fragment was also deleted from the BeninΔDP148RΔEP153R virus. We also detected three synonymous single nucleotide polymorphisms (SNPs) at genome positions 67636, 67641, and 68016 and a non-synonymous SNP at genome position 67,985 as described above. Although an HA tag was fused to the C-terminus of CD2v to facilitate detection of CD2v in infected cells (Fig. 5A), recombination occurred upstream of the tag and therefore the HA sequence was not present in either wild-type or mutant CD2v viruses.

### Multistep virus growth curves

Purified PBMs from two different pigs were infected with the ASFV Benin 97/1 or the recombinant BeninΔDP148RΔEP153R-CD2v_mutE99R at a MOI of 0.01 in triplicates. The cells and supernatants were collected at different days post-infection and subjected to three freeze*–*thaw cycles. Cellular debris was removed by centrifugation, and viral titers were obtained as described above. Repeated measure two-way ANOVA, with the Šídák’s multiple comparisons test was performed to evaluate the differences between the groups over time.

### Western blotting

Vero cells transiently expressing the wild-type or mutant CD2v were washed with PBS, and then lysed with 0.1 mL Pierce RIPA buffer (Thermo Scientific, UK) supplemented with Halt Protease Inhibitor Cocktail. Clarified cell lysates were quantified with the Pierce Coomassie (Bradford) Protein Assay Kit (Thermo Scientific). Proteins were separated on 8% Bolt Bis-Tris mini gels and transferred into Amersham Hybond P 0.45 blotting membrane (Cytiva). In some experiments, cell lysates were treated with peptide-N-glycosidase F (PNGase F) before SDS-PAGE separation. Membranes were blocked with 5% blotto (5% milk in 0.1% TBS-Tween20). Membranes were stained with rat anti-HA primary antibody followed by goat anti-rat HRP (Invitrogen). Bound antibodies were detected using ECL Select chemiluminescent detection reagent, and membranes were imaged on Syngene G: Box.

### Immunization and challenge experiments in pigs

Animal experiments were carried out in SAPO4 high containment animal housing at the Pirbright Institute according to regulated procedures from the Animals Act UK 1998 and conducted under Home Office License 7088520. All the pigs involved were female Large White Landrace crosses with initial weights of 15–22 kg. All virus inoculations and challenge were performed by the intramuscular route (IM). Six pigs were immunized (day 0) and boosted (day 21) with 10^4^ TCID_50_ BeninΔDP148RΔEP153R-CD2v_mutE99R (group B) and challenged (day 45) with 10^3^ HAD_50_ of the ASFV virulent Benin 97/1 isolate.

Clinical scoring to record temperatures, anorexia, behavior, and other signs, including vomiting, hemorrhagic diarrhea, respiratory distress, lameness, and hemorrhagic skin lesions were conducted daily (Table S2) (64). The pigs were euthanized at a moderate severity humane endpoint as defined in the project license PPL70/8852 from the UK Home Office. The moderate humane end point was typically reached following 4 days of temperature at 40.6°C or above or for 3 days if other clinical signs were observed. Other signs contributing to the end point included not eating for a second day and unwillingness to get up. At necropsy, enlargement and appearance of hemorrhages in the spleen, tonsils, and lymph nodes were recorded in addition to other signs, including lung pathology, petechiae on kidneys, fluid in the pericardium or ascites (65).

### Measurement of viral genome copies in blood

Whole blood was collected from pigs at different days after immunization and challenge. DNA from blood was extracted using the MagVet universal isolation kit (Life Technologies). The samples were assayed in duplicate for the presence of viral DNA by qPCR on a Stratagene Mx3005P system (Agilent Technologies) using primers and probes against ASFV p72 (64, 66). A standard curve was prepared from a p72 mimic plasmid by making serial dilution ranging from 10^8^ to 10^1^ copies/mL. Results were expressed as log_10_ genome copies/mL.

### ASFV specific antibody responses measured by ELISA

The sera from pigs’ blood collected at various days post-immunization and challenge were extracted via centrifugation, and the level of anti-p72 antibodies was measured using a commercial blocking ELISA (INgezim PPA Compac, Ingenasa) following the manufacturer’s instructions. The percentage of blocking (PB) was calculated using the following formula: [(negative-control OD − sample OD)/(negative-control OD − positive-control OD)] × 100, where OD is optical density. Samples were considered positive if the PB was above the cutoff value of 50% blocking.

### Cellular responses measured by IFN-γ ELISpot assay

The peripheral blood mononuclear cells (PBMCs) collected before immunization, boost, and challenge were isolated from the EDTA whole blood of the immunized pigs via gradient centrifugation on Histopaque-1083. ELISpot plates (MAIPS4510, Millipore) were coated overnight at 4°C with 4 µg/mL anti-porcine IFN-γ monoclonal antibody (P2F6, Invitrogen, ThermoFisher Scientific) diluted in 0.05 M carbonate-bicarbonate buffer. After incubation, plates were washed four times with phosphate-buffered saline (PBS). The cells were plated in duplicate at two different dilutions (8 × 10^5^ and 4 × 10^5^ per well), in RPMI-1640-Glutamax (Gibco) supplemented with 10% FBS, 50 µM 2-mercaptoethanol and 1% pen-strep. The cells were then incubated overnight for approximately 16–18 h at 37°C in a final volume of 200 µL with 10^5^ HAD_50_ of Benin 97/1, an equivalent volume of mock inoculum, or 20 µg/mL phytohemagglutinin as a positive control. The cells were then lysed by incubation for 5 min in water and then washed with PBS. Following incubations with firstly, biotinylated anti-porcine IFN-γ monoclonal antibody (P2C11, Invitrogen ThermoFisher Scientific) and subsequently, streptavidin alkaline phosphatase, the AP conjugate substrate kit (Bio-Rad), was used to develop spots. The spot-forming cells (SFC) were then counted using an ELISpot assay reader system (Immunospot, CTL), and the number of spots per well was converted into the number of spots per million cells.

## Data Availability

The authors confirm that the data supporting the findings of this study are available within the article and its supplemental materials. Any additional information will be made available on request.

## SUPPLEMENTAL MATERIAL

Figure S1 mbio.01655-24-s0001.tiff Structural analysis of CD2v.

Figure S2 mbio.01655-24-s0002.pdf Transient expression of C-terminus HA-tagged CD2v.

Figure S3 mbio.01655-24-s0003.pdf HAD in cells transiently expressing CD2v proteins.

Figure S4 mbio.01655-24-s0004.tiff Alignment of the extracellular domains of 36 CD2v proteins.

Figure S5 mbio.01655-24-s0005.pdf Transient expression of N-terminus HA-tagged CD2v proteins.

Supplemental Legends mbio.01655-24-s0006.docx Legends for Fig. S1 to S5.

Table S1 mbio.01655-24-s0007.docx Summary of animal experiments using BeninΔDP148R as parental virus.

Table S2 mbio.01655-24-s0008.docx Clinical scoring sheet.

ASM does not own the copyrights to Supplemental Material that may be linked to, or accessed through, an article. The authors have granted ASM a non-exclusive, world-wide license to publish the Supplemental Material files. Please contact the corresponding author directly for reuse.
