# Supplementary figures and images for: From structure prediction to function: defining the domain on the African swine fever virus CD2v protein required for binding to erythrocytes
Source: mBio. 2024 Dec 17;16(2):e01655-24. doi: 10.1128/mbio.01655-24 (PMC11796414; doi:10.1128/mbio.01655-24)

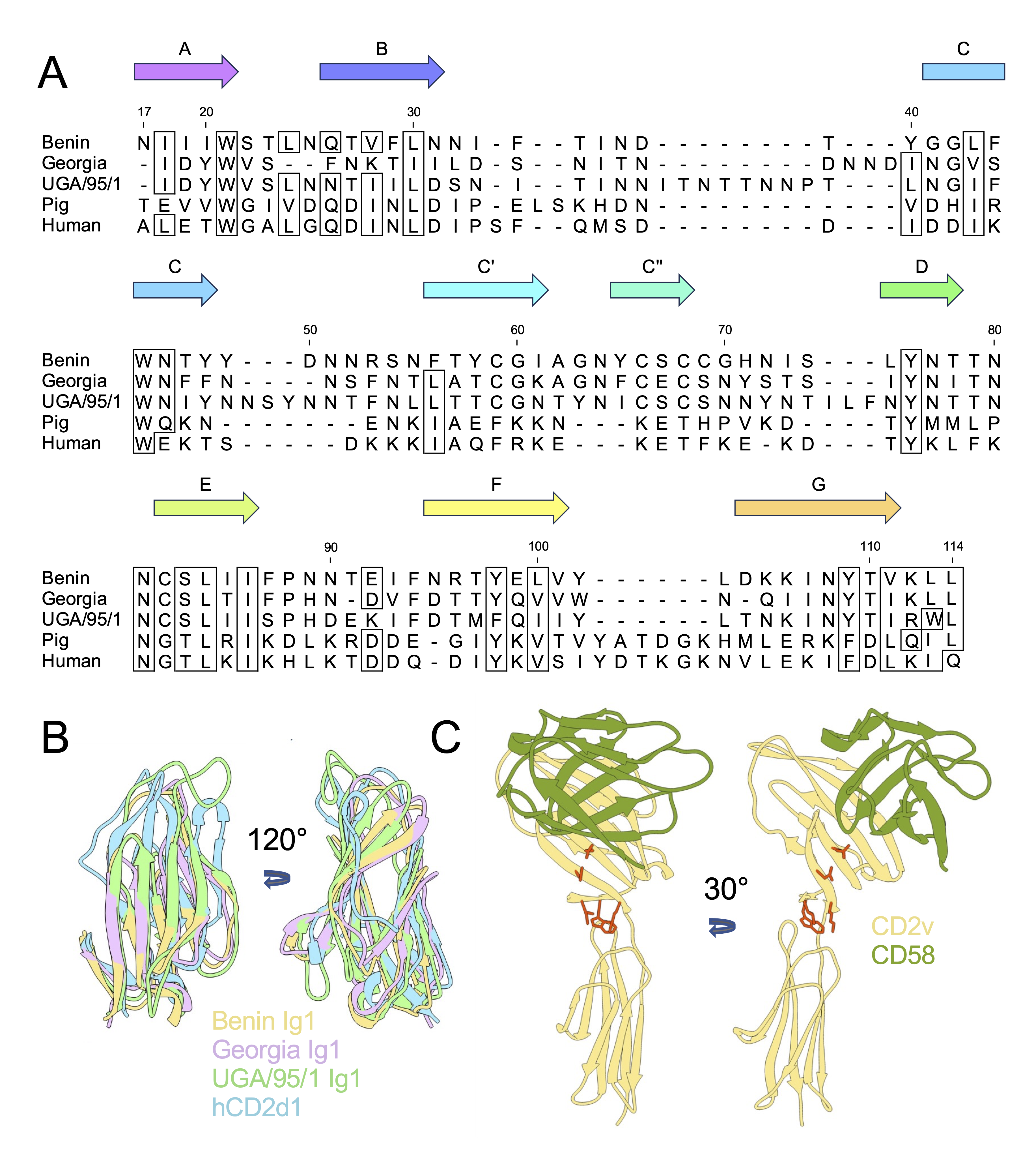

Supplement: Figure S1 — Structural analysis of CD2v. [file mbio.01655-24-s0001.tiff]

20x

63x

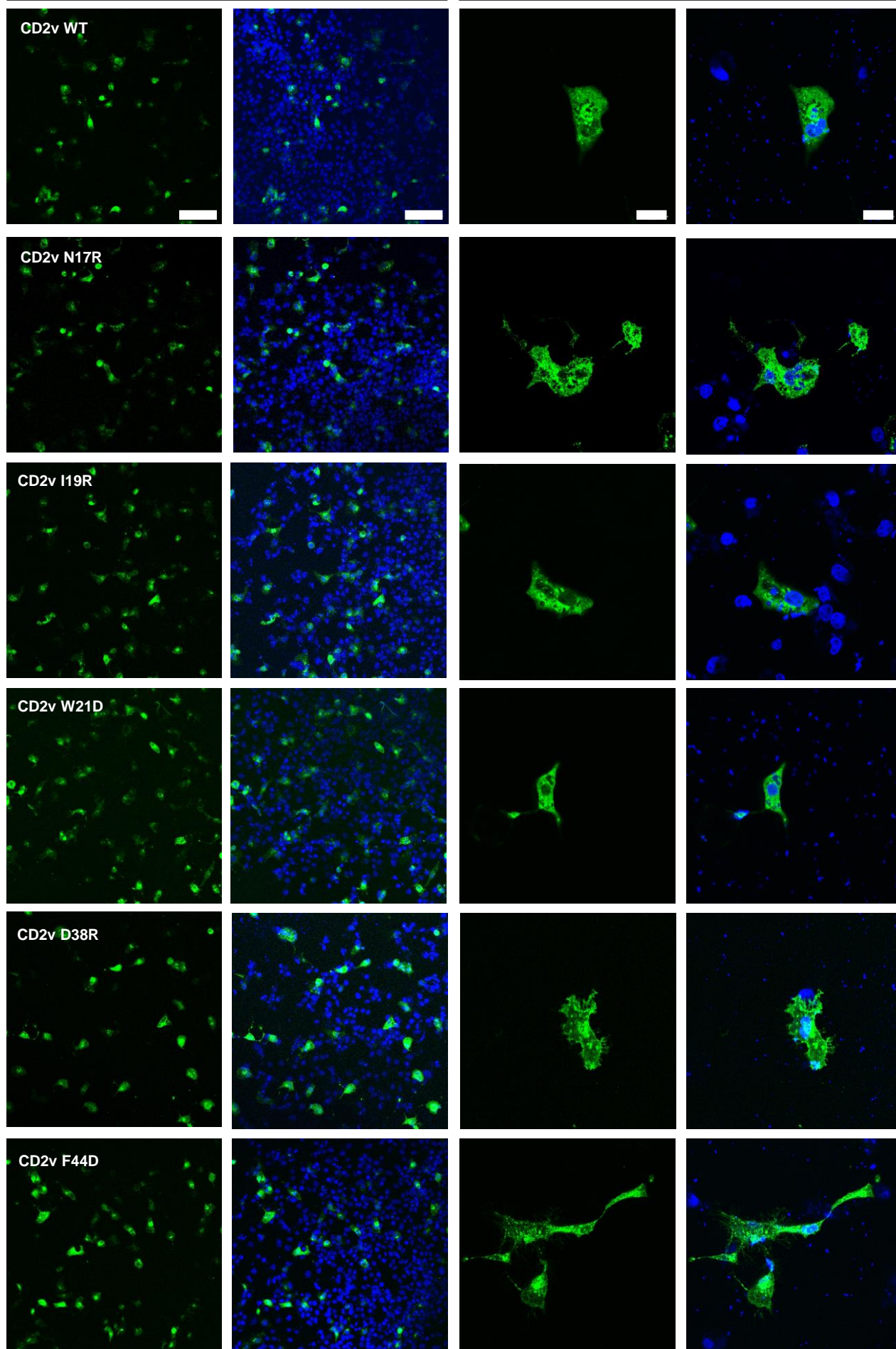

20x

63x

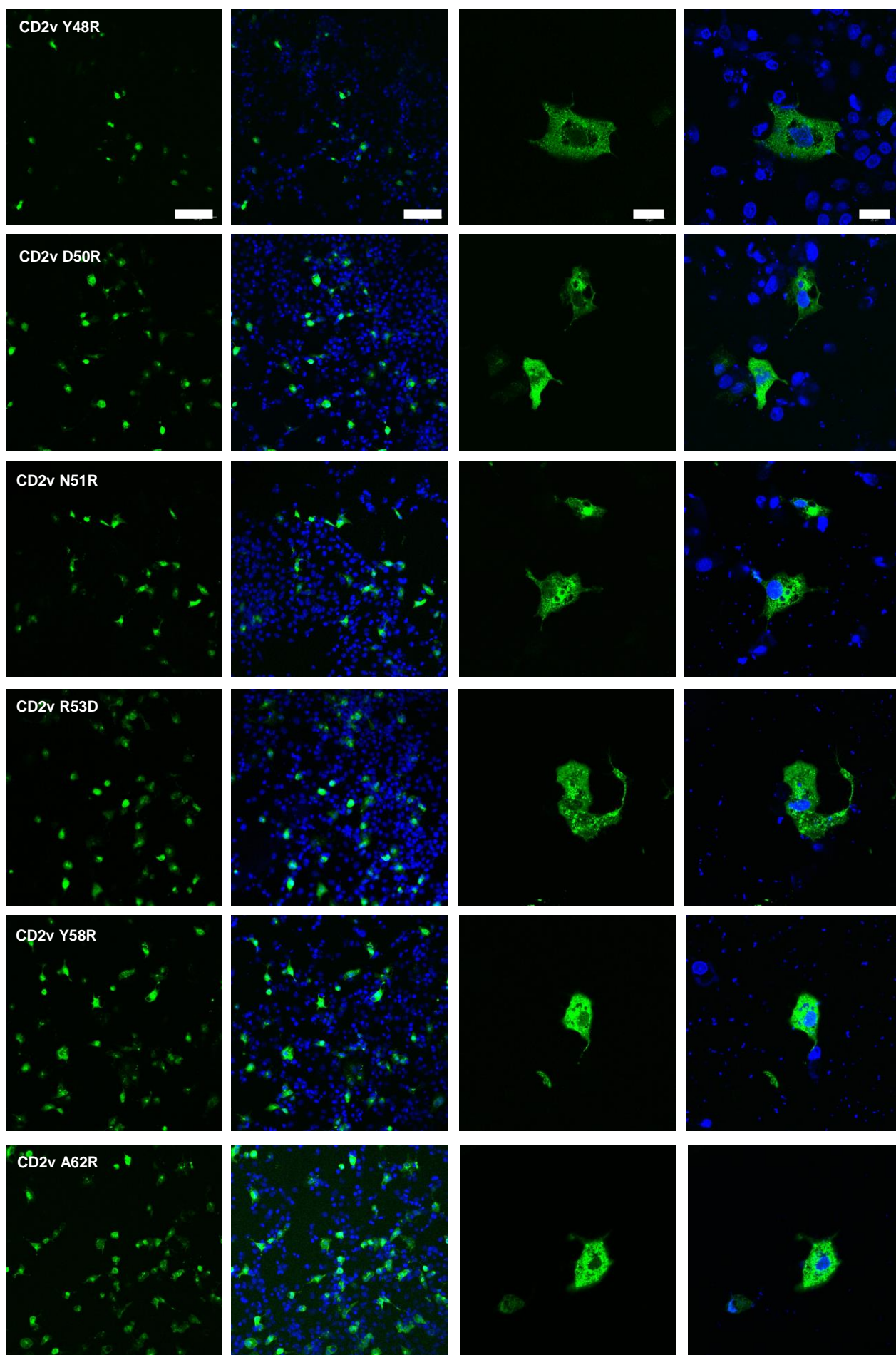

20x

63x

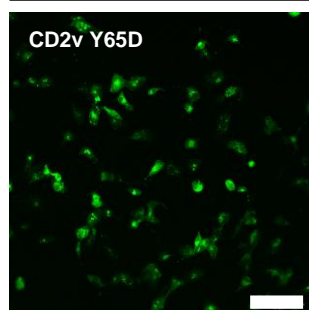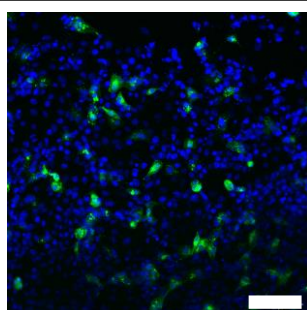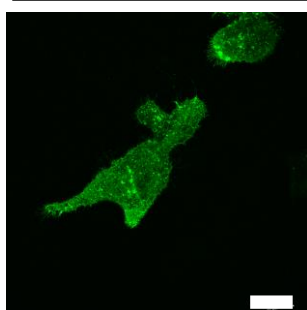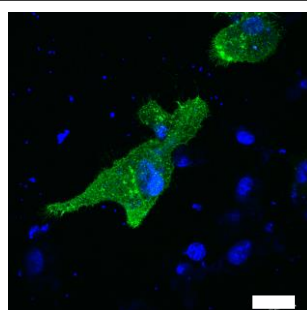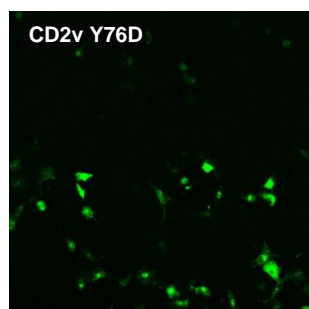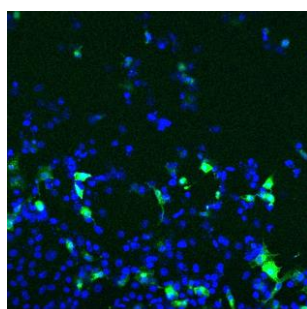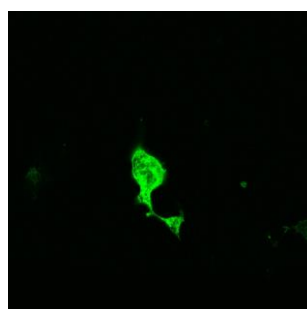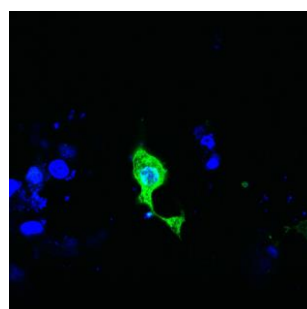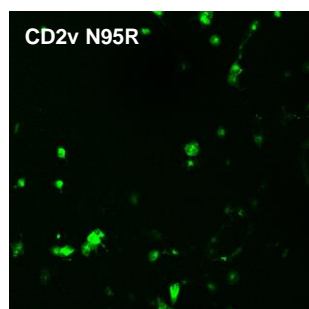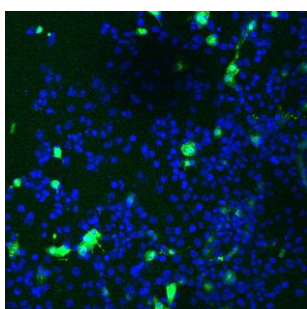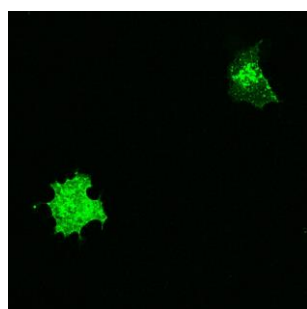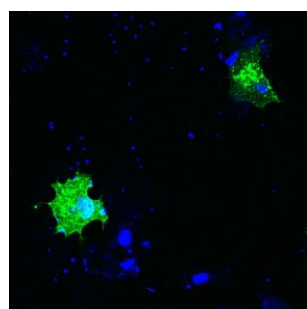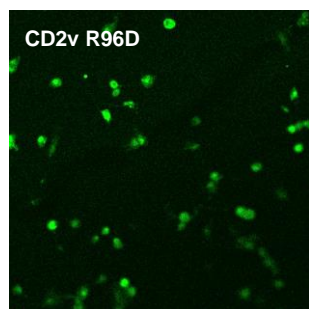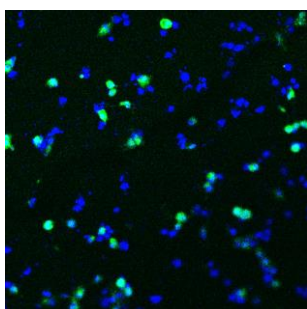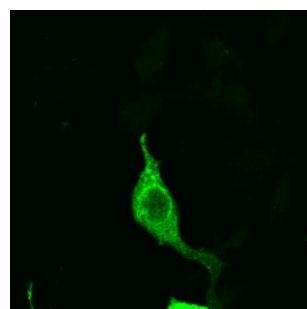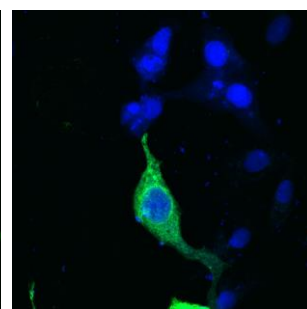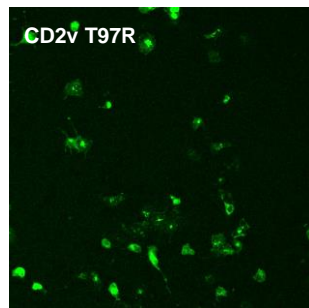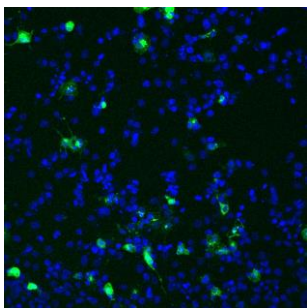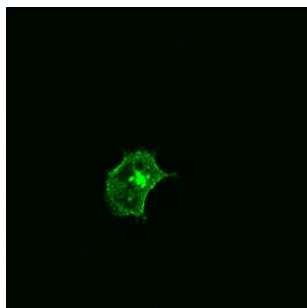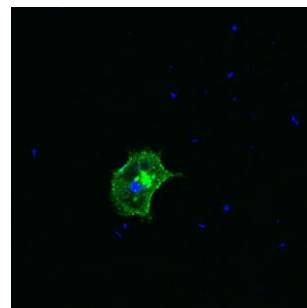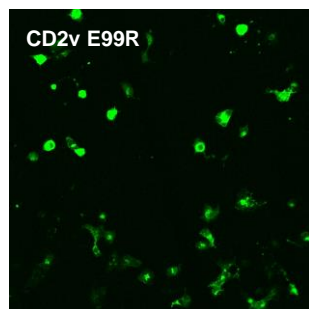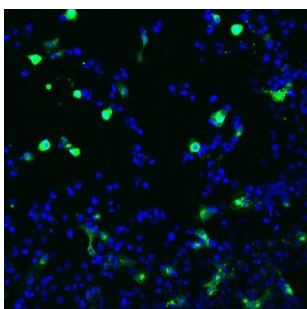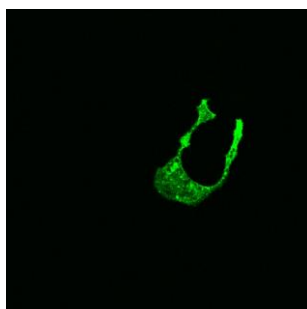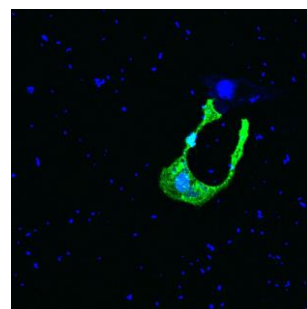

20x

63x

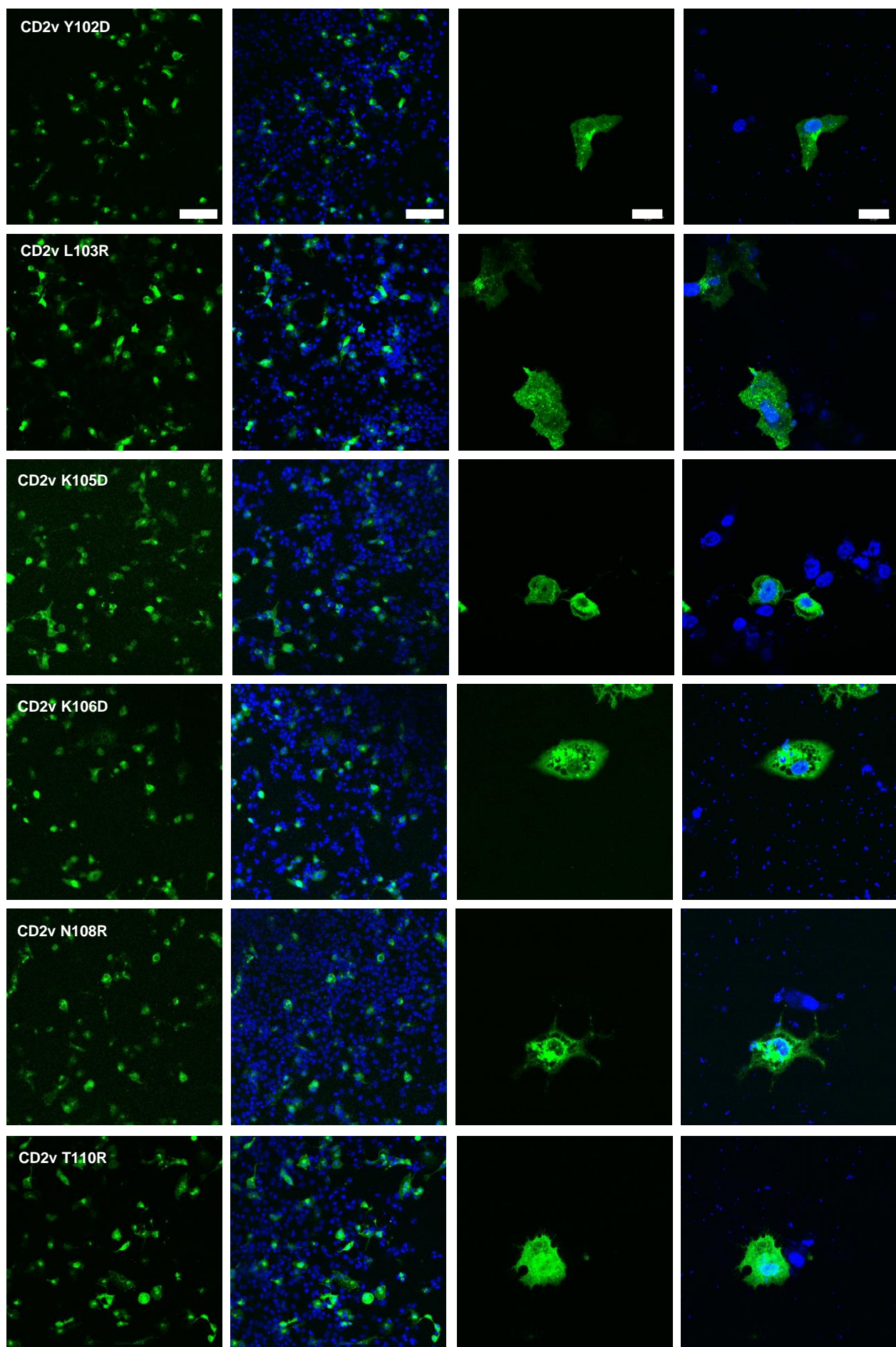

20x

63x

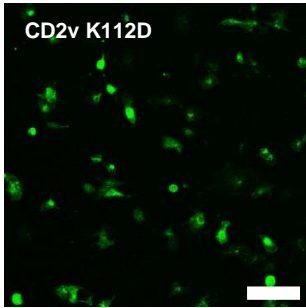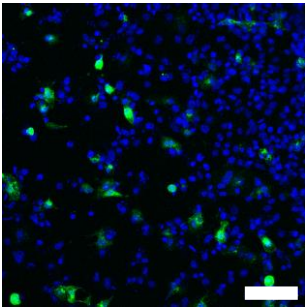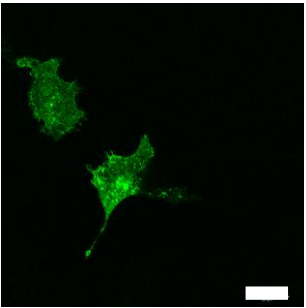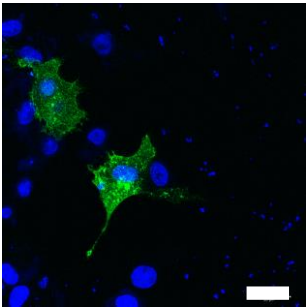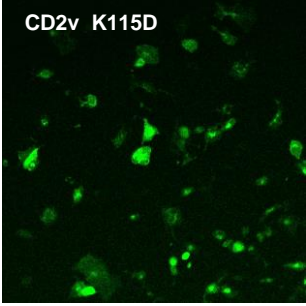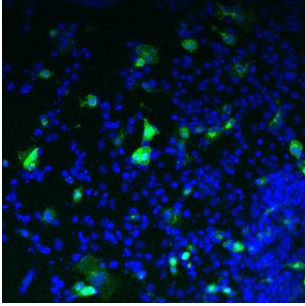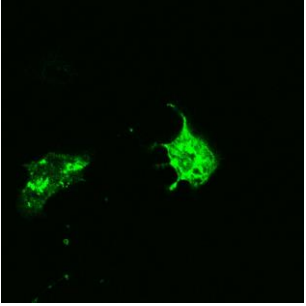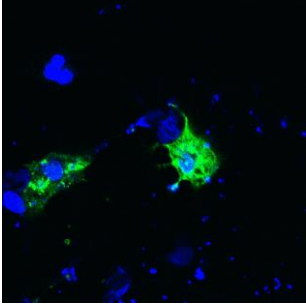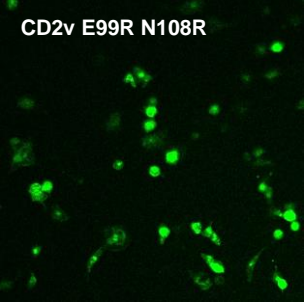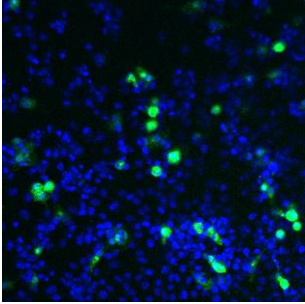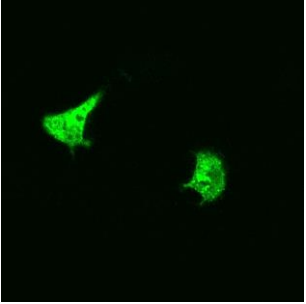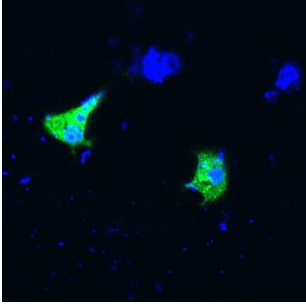

Supplement: Figure S2 — Transient expression of C-terminus HA-tagged CD2v. [file mbio.01655-24-s0002.pdf]

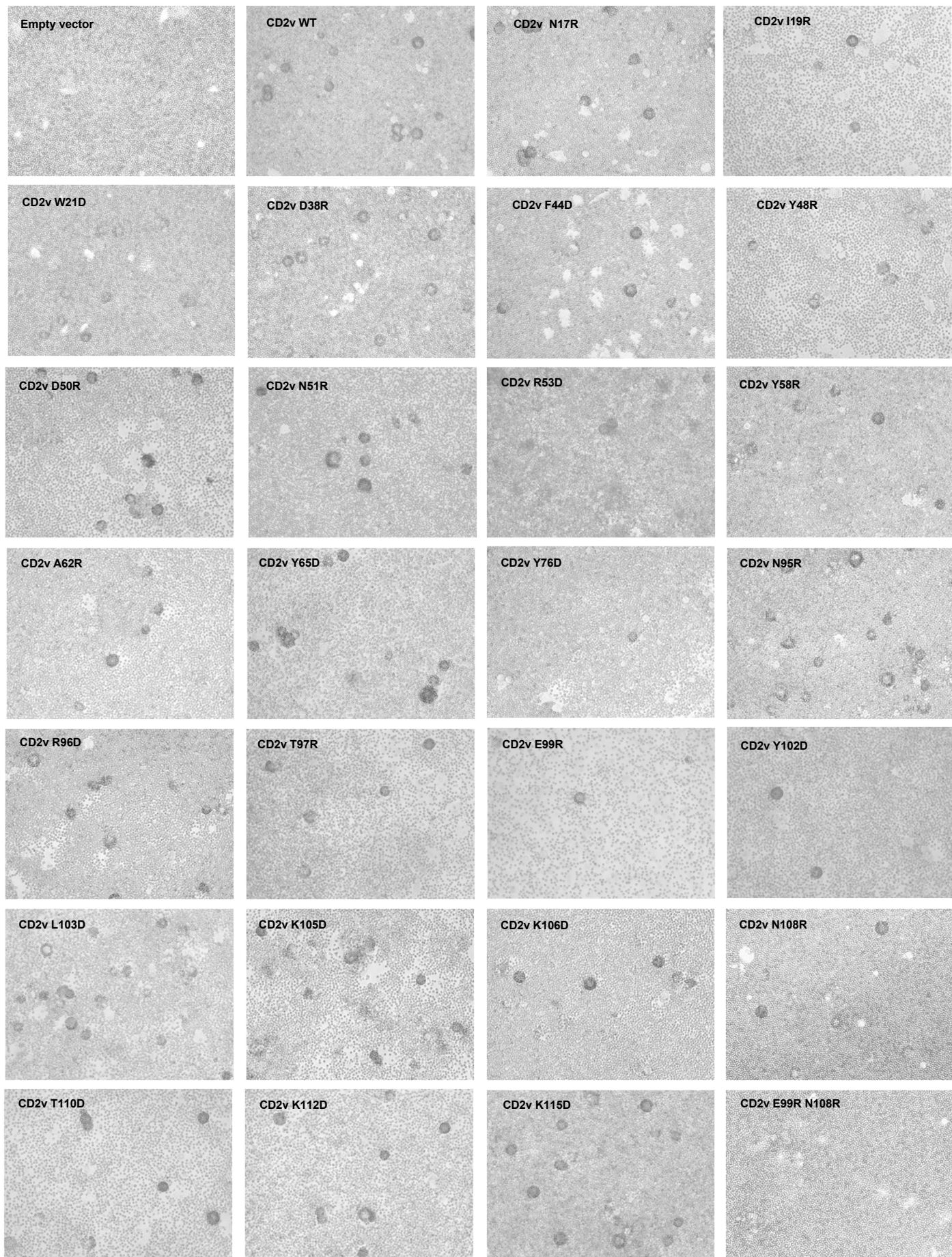

Supplement: Figure S3 — HAD in cells transiently expressing CD2v proteins. [file mbio.01655-24-s0003.pdf]

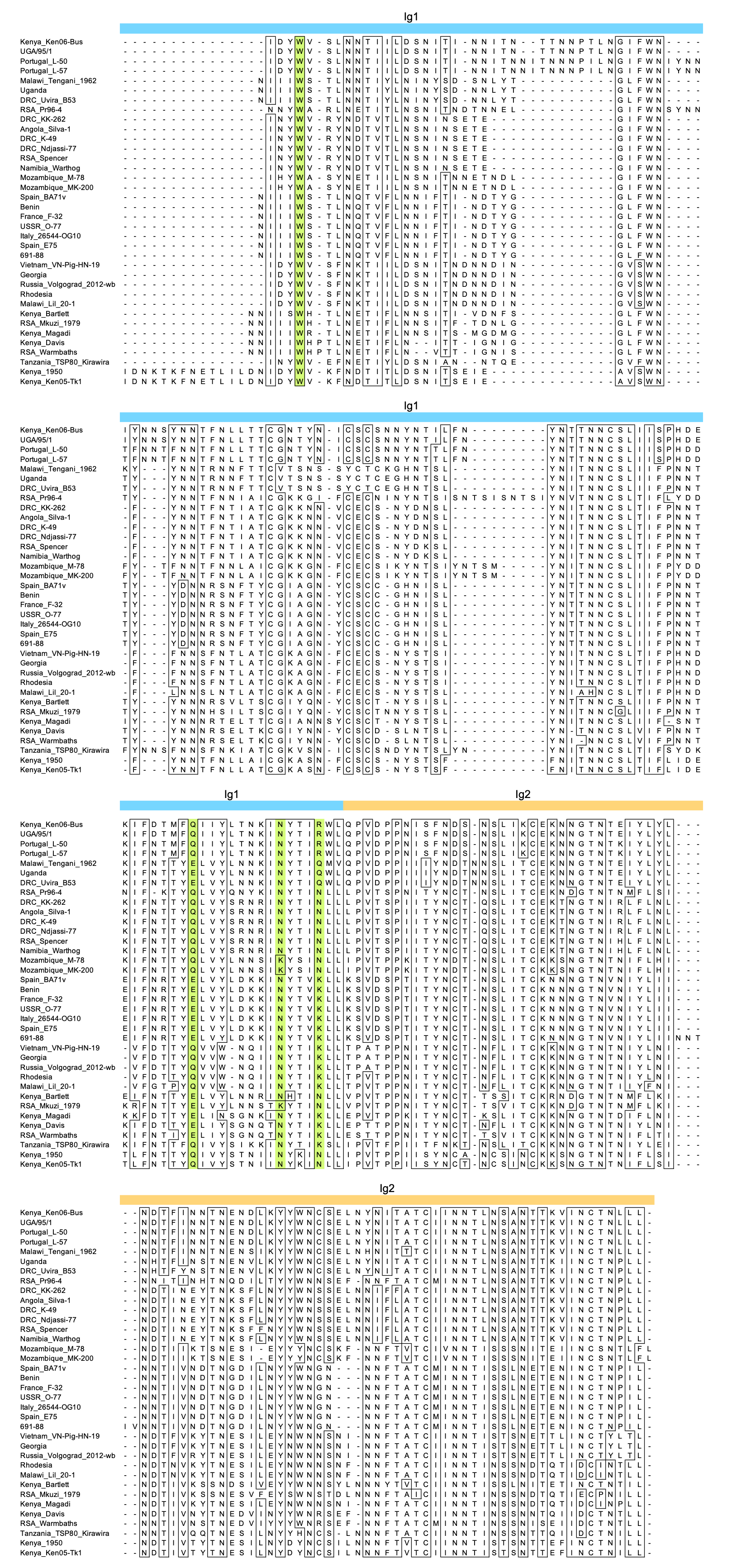

Supplement: Figure S4 — Alignment of the extracellular domains of 36 CD2v proteins. [file mbio.01655-24-s0004.tiff]

# Permeabilised

20x

63x

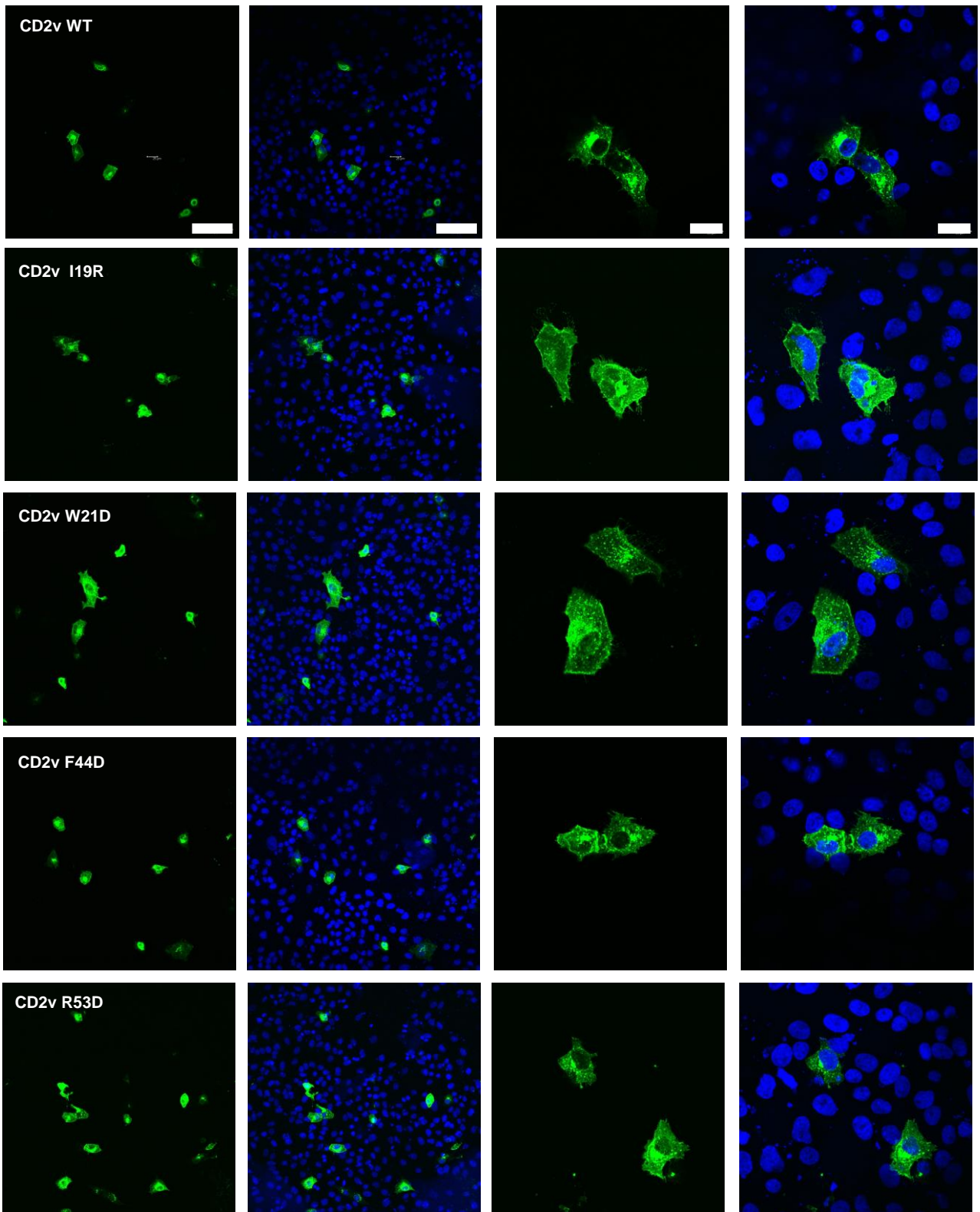

20x

63x

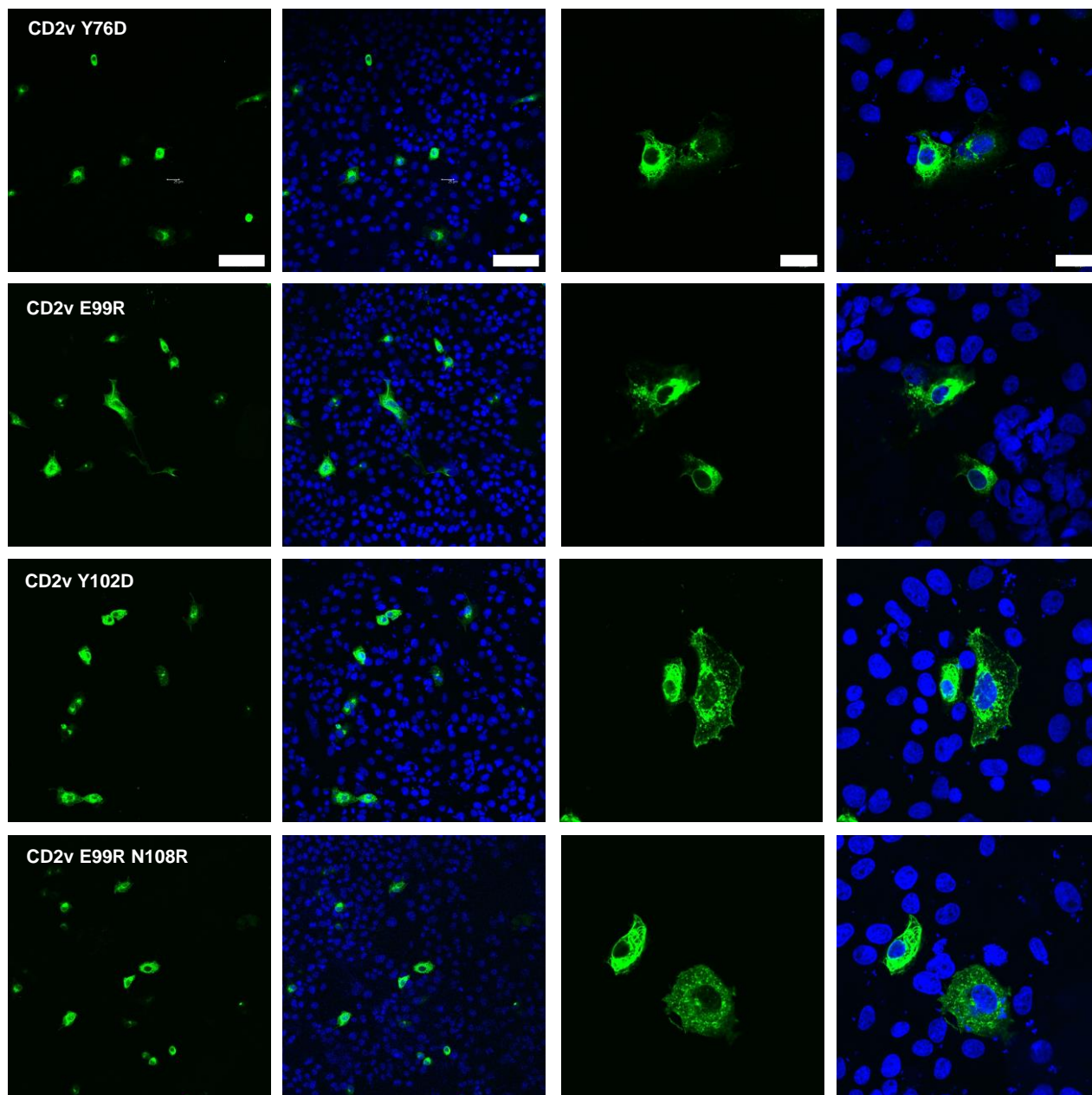

Non-permeabilised

20x

63x

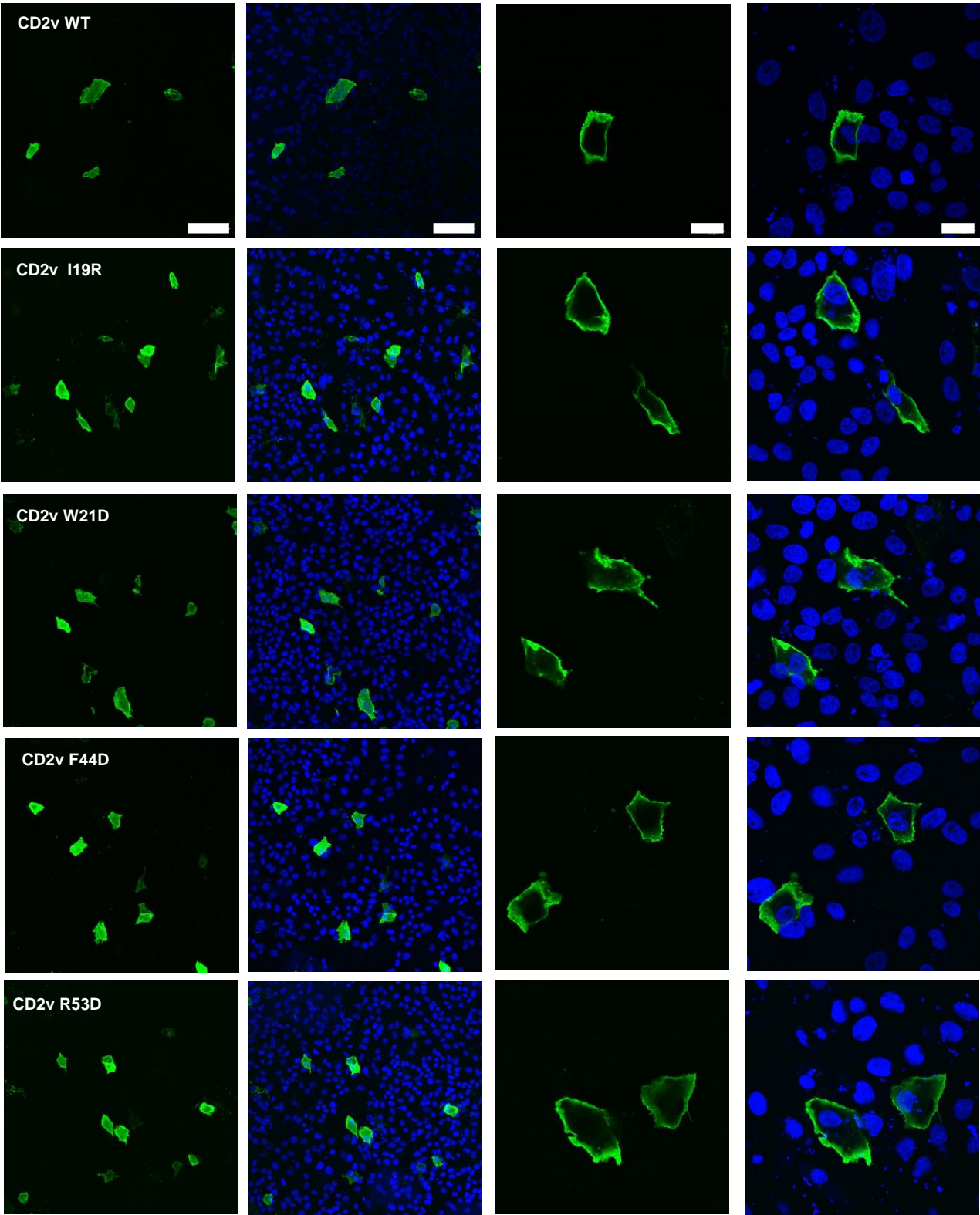

20x

63x

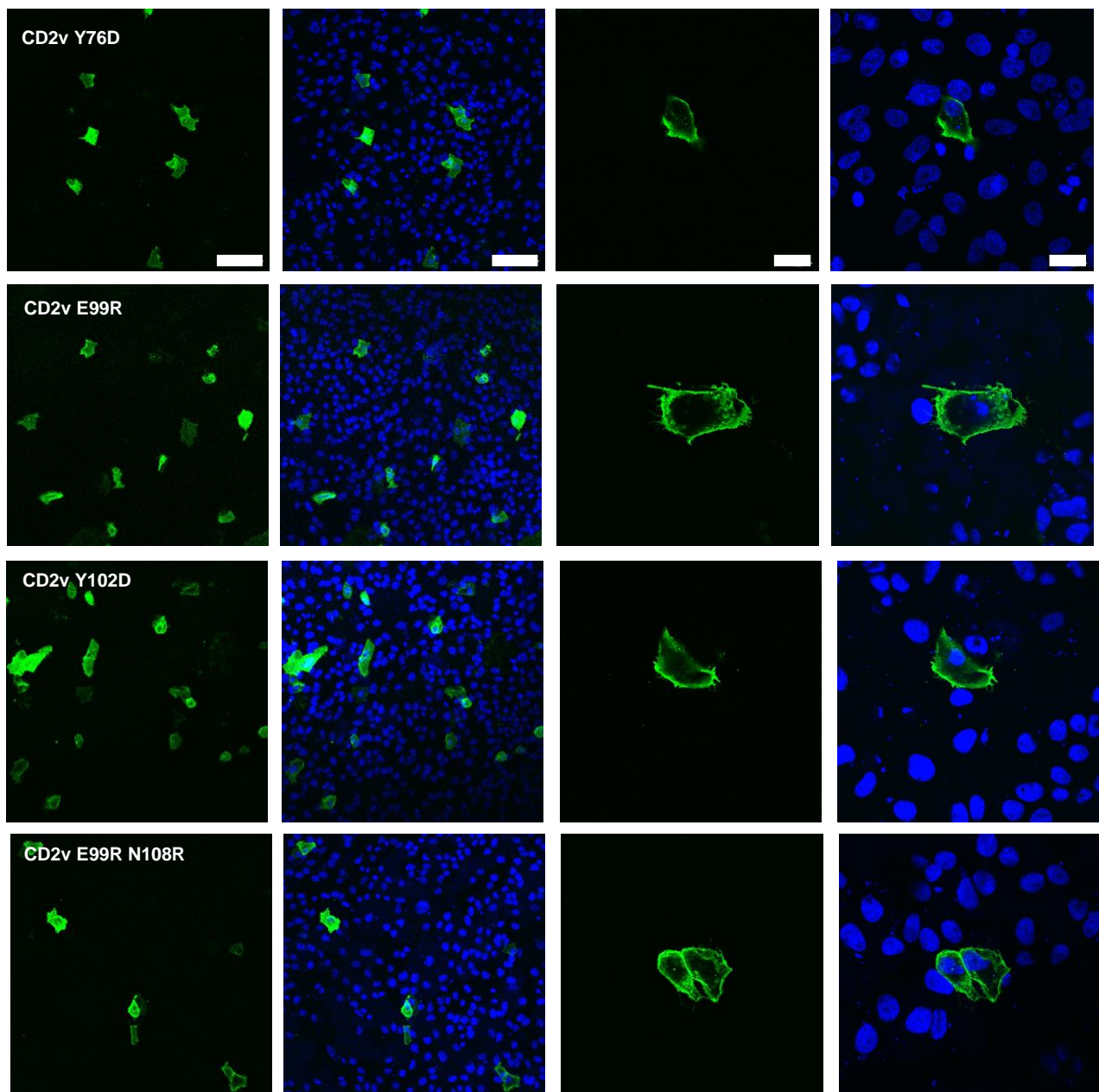

Supplement: Figure S5 — Transient expression of N-terminus HA-tagged CD2v proteins. [file mbio.01655-24-s0005.pdf]
