# Supplementary material for: From structure prediction to function: defining the domain on the African swine fever virus CD2v protein required for binding to erythrocytes
Source: mBio. 2024 Dec 17;16(2):e01655-24. doi: 10.1128/mbio.01655-24 (PMC11796414; doi:10.1128/mbio.01655-24)
Supplement: Supplemental Legends — Legends for Fig. S1 to S5. [file mbio.01655-24-s0006.docx]

**Supplementary figures legends**

**Figure S1. Structural analysis of CD2v. (A)** A structural alignment of the N-terminal Ig1 domains of the indicated CD2v proteins, and the N-terminal IgV-like domains of pig and human CD2. The Benin and Georgia CD2v and pig CD2 structures were built using AlphaFold2, and UGA/95/1 CD2v using Rosetta based on the Benin structure; the human CD2 structure was previously solved crystallographically (PDB: 1HNF). The β strands of Benin are indicated with arrows above the sequences. Residues with similar amino acid properties (i.e., [DE], [FY], [ILV], [QN], [ST], [KR], [AG]) in four or more sequences (i.e., ≥80% sequence identity) are boxed. The sequences of the proteins were from NCBI: Benin (NP_042752), Georgia (YP_009927182), UGA/95/1 (unpublished), and pig (HCZ77466). **(Β)** Ribbon representations of in silico models of Ig1 of Benin (orange), Georgia (purple) and UGA/95/1 (green) CD2v, and the crystal structure of the hCD2 V-like domain (blue), superimposed on the Benin structure. **(C)** Model of the CD2v-CD58 complex. Ribbon representation of a complex between the extracellular region of CD2v (Benin 97/1; orange) and the IgV domain of CD58 (green) based on the human CD2-CD58 complex structure (PDB: 1QA9). The CD2v residues involved in binding to erythrocytes are coloured red.

**Figure S2. Transient expression of C-terminus HA-tagged CD2v.** Vero cells were infected with MVA-T7 and transfected with plasmids expressing Benin 97/1 CD2v, wildtype (wt) or mutants with the indicated single or double amino acid substitutions. After 48h, the cells were fixed and permeabilised before probing with rat anti-HA. This was followed by incubation with goat anti-rat Alexa-Fluor 488 (green) and counterstaining with DAPI to show the nucleus (blue). Magnifications of 20x and 63x are shown, bars represent 100 µm and 25 µm respectively (shown only in the first panel for simplicity).

**Figure S3. HAD in cells transiently expressing CD2v.** Vero cells were infected with MVA-T7 and transfected with plasmids expressing Benin 97/1 CD2v, wildtype (wt) or mutants with the indicated single or double amino acid substitutions. After 48h, erythrocytes were added, and the presence or absence of rosettes (HAD) was evaluated qualitatively. Representative fields are shown (magnification of 10x).

**Figure S4. Alignment of the extracellular domains of 36 CD2v proteins.** Residues with similar amino acid properties (i.e., [DE], [FY], [ILV], [QN], [ST], [KR], and [AG]) in 29 or more sequences (i.e. ≥80% identity) are boxed. Positions of the functionally conserved residues, i.e., W21, E99, N108, and K112 are highlighted in green.

**Figure S5. Transient expression of N-terminus HA-tagged CD2v.** Vero cells were transfected with plasmids expressing codon optimised Benin 97/1 CD2v, wildtype (wt) or mutants with the indicated single or double amino acid substitutions. After 48h, the cells were fixed and either permeabilised for detection of intracellular proteins or non-permeabilised for detection of proteins at the cell surface. Cells were probed with rat anti-HA, followed by goat anti-rat Alexa-Fluor 488 (green) and counterstaining with DAPI to show the nucleus (blue). Magnifications of 20x and 63x are shown, bars represent 100 µm and 25 µm respectively (shown only in the first panel for simplicity).
