# Supplementary material for: From structure prediction to function: defining the domain on the African swine fever virus CD2v protein required for binding to erythrocytes
Source: mBio. 2024 Dec 17;16(2):e01655-24. doi: 10.1128/mbio.01655-24 (PMC11796414; doi:10.1128/mbio.01655-24)
Supplement: Table S1 — Summary of animal experiments using BeninΔDP148R as parental virus. [file mbio.01655-24-s0007.docx]

**Table S1: Summary of animal experiments using BeninΔDP148R as parental virus.**

| **Recombinant ASFV** | **Dose** | | **Post-immunisation** | | | |  | **Protection**** |
| --- | --- | --- | --- | --- | --- | --- | --- | --- |
|  |  |  | **>40.5°C** | **Other signs** | **Viremia** | | |  |
|  | **Prime** | **Boost** | **No. pigs** | **No. pigs** | **No. pigs** | **Peak (Log)*** | **Duration (dpi)** |  |
| BeninΔDP148R^1^ | 10^3^ | 10^3^ | 5/5 | 0/5 | 5/5 | 6.2 | 4 to 59 | 100.0% |
| BeninΔDP148R^2^ | 10^5^ | 10^5^ | 5/5 | 4/5 | 5/5 | 6.3 | 4 to 60 | 100.0% |
| BeninΔDP148RΔCD2v^2^ | 10^3^ | 10^4^ | 4/4 | 4/4 | 4/4 | 4.7 | 5 to 14 | 100.0% |
| BeninΔDP148RΔEP153R^2^ | 10^5^ | 10^5^ | 6/6 | 6/6 | 6/6 | 7.2 | 3 to 60 | 100.0% |
| BeninΔDP148RΔEP153RΔCD2v^2^ | 10^4^ | 10^4^, 10^6^ | 0/8 | 0/8 | 0/8 | Not detected | Not detected | 75.0% |
| BeninΔDP148RΔEP153R-CD2v_mutantE99R | 10^4^ | 10^4^ | 3/6 | 0/6 | 4/6 | 3.1 | 6 | 83.3% |

*Peak (log) refers to peak genome copies per mL blood of the recombinant virus detected via qPCR. ** Protection refers to protection afforded by recombinant viruses against genotype I, Benin 97/1 virulent challenge. Data in the table are from the following studies ^1^ Reis et al., 2017 and ^2^ Petrovan et al. 2022.
