# Supplementary material for: From structure prediction to function: defining the domain on the African swine fever virus CD2v protein required for binding to erythrocytes
Source: mBio. 2024 Dec 17;16(2):e01655-24. doi: 10.1128/mbio.01655-24 (PMC11796414; doi:10.1128/mbio.01655-24)
Supplement: Table S2 — Clinical scoring sheet. [file mbio.01655-24-s0008.docx]

| **Score** | **0** | **1** | **2** | **3** | **4** | **5** | **6** |
| --- | --- | --- | --- | --- | --- | --- | --- |
| **Temperature** | <39.0 | 39.0 - 39.5 | 39.6 - 40.0 | 40.1- 40.5 | 40.6 - 41.0 | >41 |  |
| **Anorexia** |  | Reduced eating |  |  | Only picking at food |  | Not eating |
| **Behaviour** |  | Lethargic | Get up only when touched |  | Slow to get up when touched |  | Remain recumbent when touched; head hung/back arched |
| **Lameness** |  | Joint swelling |  |  | Severe swelling with difficulty walking |  |  |
| **Respiratory**  **system** |  | Laboured/increase breathing and/or coughing |  |  | Severe respiratory signs |  |  |
| **Faeces/Urine** |  | Diarrhoea |  |  | Bloody diarrhoea or blood in urine |  |  |
| **Vomiting** |  |  |  |  | Vomiting |  |  |
| **Haemorrhage** |  | Haemorrhage on ears and body |  |  | Generalized haemorrhage all over body |  |  |
| **Discharges** |  | Ocular discharge |  |  |  |  |  |

**Table S2. ASF clinical scoring (adapted from Galindo-Cardiel et al., 2013)**
